# Advances in Laser Drilling of Structural Ceramics

**DOI:** 10.3390/nano12020230

**Published:** 2022-01-11

**Authors:** Xianshi Jia, Yongqian Chen, Lei Liu, Cong Wang, Ji’an Duan

**Affiliations:** 1 State Key Laboratory of High Performance and Complex Manufacturing, College of Mechanical and Electrical Engineering, Central South University, Changsha 410083, China; leiliu2020@csu.edu.cn (L.L.); wangcong@csu.edu.cn (C.W.); duanjian@csu.edu.cn (J.D.); 2 School of Mechatronics Engineering, Zhongyuan University of Technology, Zhengzhou 450007, China; chenyq@zut.edu.cn

**Keywords:** structural ceramic, laser drilling, millisecond laser, nanosecond laser, picosecond laser, femtosecond laser, combined pulse laser

## Abstract

The high-quality, high-efficiency micro-hole drilling of structural ceramics to improve the thermal conductivity of hot-end parts or achieve high-density electronic packaging is still a technical challenge for conventional processing techniques. Recently, the laser drilling method (LDM) has become the preferred processing tool for structural ceramics, and it plays an irreplaceable role in the industrialized processing of group holes on structural ceramic surfaces. A variety of LDMs such as long pulsed laser drilling, short pulsed laser drilling, ultrafast pulsed laser drilling, liquid-assisted laser drilling, combined pulse laser drilling have been developed to achieved high-quality and high-efficiency micro-hole drilling through controlling the laser–matter interaction. This article reviews the characteristics of different LDMs and systematically compares the morphology, diameter, circularity, taper angle, cross-section, heat affect zone, recast layer, cracks, roughness, micro–nano structure, photothermal effect and photochemical reaction of the drilling. Additionally, exactly what processing parameters and ambient environments are optimal for precise and efficient laser drilling and their recent advancements were analyzed. Finally, a summary and outlook of the LDM technology are also highlighted.

## 1. Introduction

The trend of product miniaturization, as well as the trend of manufacturing miniature functional parts and components, require new tools and manufacturing methods [1]. Among these, high-aspect-ratio (>5:1) micro-holes have become one of the typical features of products, such as the lab-on-a-chip which uses micro-channel networks to transport, mix, separate, and detect samples; the important guiding structure for the gas/medium that is used in the ignition target ball and micro-heat exchanger; the cooling holes in the aeroengine turbines blades [2,3,4]. On the other hand, the rapid development of device miniaturization in recent years has promoted the high-density interconnection of electronic systems. Multi-size, multi-pitch, and high-density micro-holes processing is used to provide internal circuit interconnection, and the processing quality needs to meet the packaging requirements of chip conduction and pin fixation [5,6,7]. In particular, such arrays of holes as the core structure of many micro-devices and systems, the processing quality, accuracy, and repeatability directly determine the performance (such as the detection sensitivity and heat dissipation performance) and integration level of the entire device/system, which introduces further constraints and thus limit the available options for their cost-effective manufacture.

Ceramics, as a typical structural and engineering material, have the characteristics of high hardness, high thermal conductivity, high resistivity, high thermal stability, etc., and have become the first choice for the new generation of microelectronic devices and systems which have been widely used in in aerospace, 5G communications, automotive applications, high-power LED lighting, and high-power power semiconductors. Conventional processing methods can easily cause the substrate to break since the ceramic is a typical hard and brittle material, and the special processing methods such as chemical etching and ultrasonic processing have also not been widely adopted due to a variety of limiting factors [8]. With the maturity and popularization of industrial laser technology, the laser processing method has become a new choice for drilling micro-holes in structural ceramics [9,10].

There have recently been a lot of studies on the laser drilling of structural ceramics, involving the processing of conventional ceramic materials such as alumina (Al_2_O_3_) ceramics [11], silicon nitride (Si_3_N_4_) ceramics [12], silicon carbide (SiC) ceramics [13], zirconia (ZrO_2_) ceramic [14], aluminum nitride (AlN) ceramics [15], and yttria-stabilized zirconia (YSZ) [16], as well as new composite ceramic materials such as zirconia-toughened alumina (ZTA) composites [17], Al-doped SiCN (SiAlCN) composites [18], (2.5 D) carbon fiber-reinforced silicon carbide (C_f_/SiC) composites [19], SiC/SiC composites [20], and titanium nitride–alumina (TiN–Al_2_O_3_) [21]; moreover, the types of laser used for drilling can be continuous wave (CW) laser, millisecond laser, microsecond laser, nanosecond laser, picosecond laser, and femtosecond laser, whilst the wavelength of the laser can also be ultraviolet, green, near infrared, etc.; in addition, the drilling method can be selected as either percussion drilling, trepan drilling, and helical drilling; moreover, the drilling can also be enhanced by the assistance of the vacuum, liquid, and combined pulse laser (CPL). Therefore, it is necessary to systematically summarize and analyze the latest progress of the laser drilling of structural ceramics.

Here, we will review the achievements and outline the current trends in the development of the laser drilling of structural ceramics from the aspects of characteristics of laser drilling, long pulsed laser drilling of ceramic, short pulsed laser drilling of ceramic, ultrafast pulsed laser drilling of ceramic, the liquid-assisted laser drilling of ceramic, and CPL drilling of ceramic. Basis upon this, by systematically comparing the morphology, diameter, taper angle, cross-section, cracks, roughness, micro–nano structure, photothermal effect, and photochemical reaction of the drilling, the drilling mechanism under the different lasers and different drilling methods are revealed. Additionally, the energy coupling between the laser and target, phase change of the matter, process of the material removal, mechanism of defect formation and inhibition, and formation of microstructure are discussed in depth. Finally, the opportunities and challenges of the laser drilling of structural ceramics are discussed.

## 2. Characteristics of Laser Drilling of Ceramics

Laser drilling is an effective way to drill holes in structural ceramics since the focused laser beam with extremely high-energy density is able to ablate the ceramics; however, it usually causes defects such as cracks and a recast layer. The hole quality is the key issue that should be carefully and comprehensively considered when different kinds of laser are used to perform the drilling. Usually, in order to characterize the hole quality of the laser drilling, multiple quality factors need to be considered, such as the morphology, diameter, hole depth, taper angle, cross-section profile, heat-affected zone (HAZ), recast layer, cracks, roughness, micro–nano structure, photothermal effect, and photochemical reaction.

**Figure 1:**
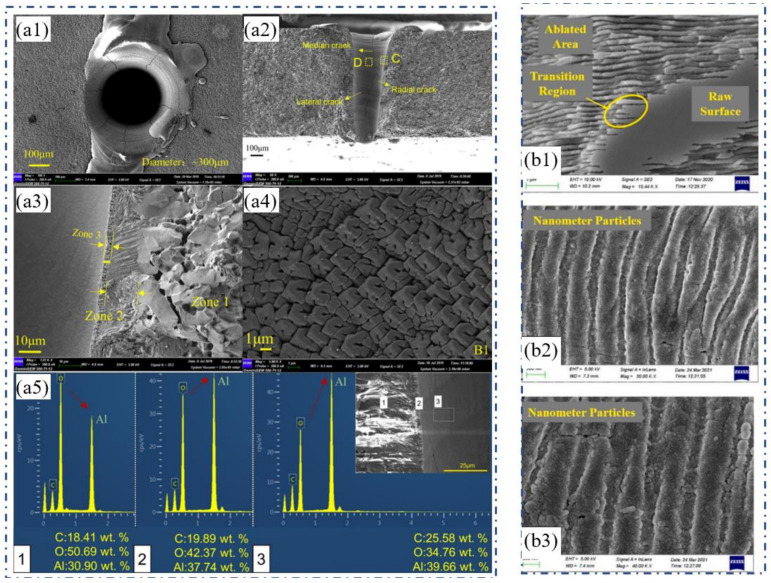
(**a1**) The surface morphology of the hole, including diameter and cracks; (**a2**) the cross-section of the hole, including taper angle, cracks, recast layer; (**a3**) the cross-section of area C in (**a2**); (**a4**) structure of the crystal; (**a5**) EDS results of cross-section of the hole (area 1 is the over-grown grains zone, area 2 is the recast layer zone, and area 3 is the re-solidified particles zone), showing a photochemical reaction of the drilling. Reprinted with permission from ref. [23] Copyright 2021 Elsevier. Femtosecond laser-induced periodic surface structure morphology (**b1**) in air; (**b2**) in water; (**b3**) in HF. Reprinted with permission from ref. [22]. Copyright 2020 Elsevier.

Figure 1a1–a5 show the common morphology of holes in the 1 mm Al_2_O_3_ substrate drilled by CPL. Since the target will absorb the laser energy which then goes through the processes of melting, vaporization, sputtering, and removal, this will lead to the formation of the recast layer, cracks, HAZ, and spatter. As shown in Figure 1a1, the hole diameter can be measured to be ~300 µm with a circularity of ~0.96, and there is spatter with a width of ~140 µm in the upper surface of the hole which is due to the cooling of the molten alumina ceramic. Figure 1a2 shows the cross-section of the hole. The taper angle is calculated to be ~3.0°, and three types of cracks are evidenced in the figure. Additionally, a local magnified SEM micrograph of the cross-section of area C in Figure 1a2 is shown in Figure 1a3, where the hole cross-section shows three different layers which are defined as zone 1 (the over-grown zone), zone 2 (the recast layer), and zone 3 (the re-solidified particle layer). Figure 1a4 shows the columnar crystal structure created by the regrowing of the recast layer adhered to the hole wall during the drilling, which has a grain size of approximately 2–3 µm with a square shape. In addition, it can be seen that in Figure 1a5, the atomic ratio of Al–O in the re-solidified particles zone is highest, and the atomic ratio of Al–O in the over-grown grains zone is the lowest. The alumina dissociates after laser drilling and causes massive oxygen vacancies, and the oxygen vacancies will cause alumina discoloration, which gives the recast layer and re-solidified particles a darker color. In addition, during femtosecond laser processing, a phenomenon that is closely related to the laser-induced periodic surface structure (LIPSS) is observed as shown in Figure 1b1–b3 [22].

In addition, laser drilling involves lots of parameters such as the processing environment, focal plane position, laser wavelength, repetition rate, pulse shape, peak power, pulse duration, and drilling time, which should be controlled in order to obtain the desired hole characteristics. Additionally, the drilling method may be selected from either percussion drilling, trepan drilling, and helical drilling. It is necessary to select the appropriate drilling method according to the actual needs since each drilling method has its own unique advantages and disadvantages. For example, the percussion drilling method is always used in millisecond laser drilling because the high-energy injected by the millisecond laser will lead to the emergence of a large amount of melt, which can be effectively removed under the ejection of the subsequent laser pulse. This kind of drilling method often has a high material removal rate (MRR), but the processing quality is average. On the other hand, the trepan drilling method is always used in femtosecond laser drilling because the small focus spot of the femtosecond laser needs to be continuously moved to ablate the whole target, showing excellent drilling quality and a low MRR.

## 3. Long Pulsed Laser Drilling of Ceramic

### 3.1. Drilling Mechanism

**Figure 2:**
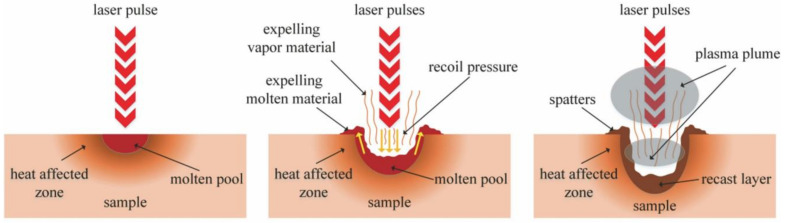
Schematic of the target drilled by the long pulsed laser [24].

The removal process of the ceramic under the irradiation of the long pulsed laser (including the millisecond laser, microsecond laser, CW laser) is mainly divided into three stages, as shown in Figure 2. First, the laser energy is absorbed and accumulated inside the target. The material melts and the molten pool is formed when its temperature reaches the melting point. The molten state of the material will increase the absorptivity of the laser. At the same time, the increase in temperature vaporizes the material, and the recoil pressure caused by the vaporization will expel the molten material, i.e., the second stage. It should be noted here that this stage is the main process of millisecond laser drilling, which is a stable process of hole formation. In the third stage, the laser energy induces plasma and forms a shielding effect on the laser transmission, hinders the transmission of laser energy into the hole, and hinders the discharge of gas and liquid substances out of the hole.

**Figure 3:**
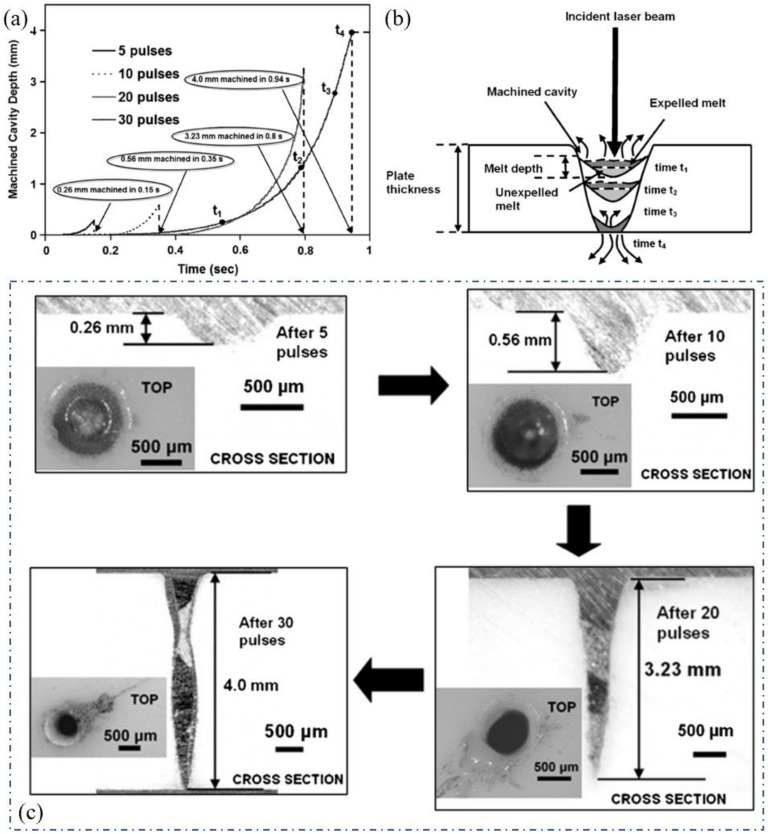
The drilling process of the ceramic: (**a**) theoretical calculation; (**b**) schematic diagram of the drilling process; and (**c**) hole drilled in alumina with different numbers of pulses (5 pulses, 10 pulses, 20 pulses and 30 pulses). Reprinted with permission from ref. [25]. Copyright 2008 Elsevier.

In order to explore the mechanism of the millisecond laser drilling of ceramics, Samant and Dahotre showed a one-dimensional hydrodynamic machining theoretical model [25,26,27]. The model comprehensively considered the influence of multiple reflections of the hole wall on laser energy, the thermal effects of the drilling, recoil pressure, the energy loss caused by vaporization, recoil pressure, and defocus caused by laser transmission in the hole. As shown in Figure 3a, before time t_1_, a keyhole was formed on the initial surface under the irradiation of laser pulses. With the processes of melting, vaporization, saturated vapor pressure, recoil pressure and expelling, the target was mainly ablated through upward vaporization and expelling; with the further increase in the hole depth, the melt not expelled in time gradually accumulated before time t_2_, which was also the main reason for the induced recast layer and cracks of the hole; At t_3_, the lower surface of the hole has melted through, and only a layer of melt remained in the hole. Because there was no solid alumina ceramic substrate at the hole lower surface, the recoil pressure had no support point, so most of the material was pushed out from the lower surface; finally, the whole through hole was formed at t_4_. This model is helpful to select the laser energy required for drilling and predicting the drilling efficiency, which also helps one to understand the dynamic drilling process. Figure 3c shows the cross-section of the drilled hole (1064 nm wavelength, 4 J pulse energy, 20 Hz repetition rate, 0.5 ms pulse duration) with different pulse numbers. Thirty pulses of the millisecond laser were able drill through the 4 mm target. The authors suggested that even the original alumina surface had a low absorptivity of the 1064 nm, and the effective absorptivity of the material could be increased due to the multiple reflections of the hole wall and three different values of absorptivity of 0.8, 0.9 and 1 were used to compare the corresponding experimental results [25].

**Figure 4:**
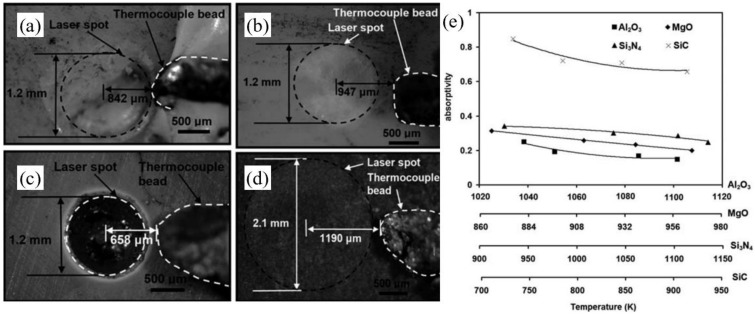
Thermocouple glued to the ceramic surface in (**a**) Al_2_O_3_, (**b**) MgO, (**c**) Si_3_N_4_, and (**d**) SiC; and (**e**) variation of the laser absorptivity of different ceramics in function of temperature.

Paying attention to the difference between the theoretically calculated absorptivity and the actual value during the drilling of alumina ceramic, Samant et al. suggested that it might be caused by the low aspect ratio machining of the ceramic in the early stage of micro-hole drilling. In light of this, during the drilling of the low aspect ratio hole, in situ temperatures sensed by a K-type thermocouple were used to calibrated the actual absorptivity by adopting a thermal model. Figure 4e shows the variation of the absorptivity of different ceramics in function of temperature. For the 1.06 µm wavelength, it was observed that the absorptivity of all structural ceramics decreased with the increase in temperature. The maximum absorptivity for Al_2_O_3_ was 0.25 at 1039 K, which was far less than the assumed absorptivity of 1. Therefore, the low aspect ratio drilling did show a low absorptivity, which was completely different from the absorptivity close to 1 under multiple reflections during the deep hole drilling [28]. Additionally, it is obvious that the absorptivity of the SiC ceramic was the highest. In this context, Samant et al. further used the theoretical model to analyze the physical phenomena underlying the drilling of SiC [29]. The material removal mechanisms of the laser drilling were summarized in Table 1 below, and a combination of melting, dissociation, and evaporation contributed to the drilling [30].

**Figure 5:**
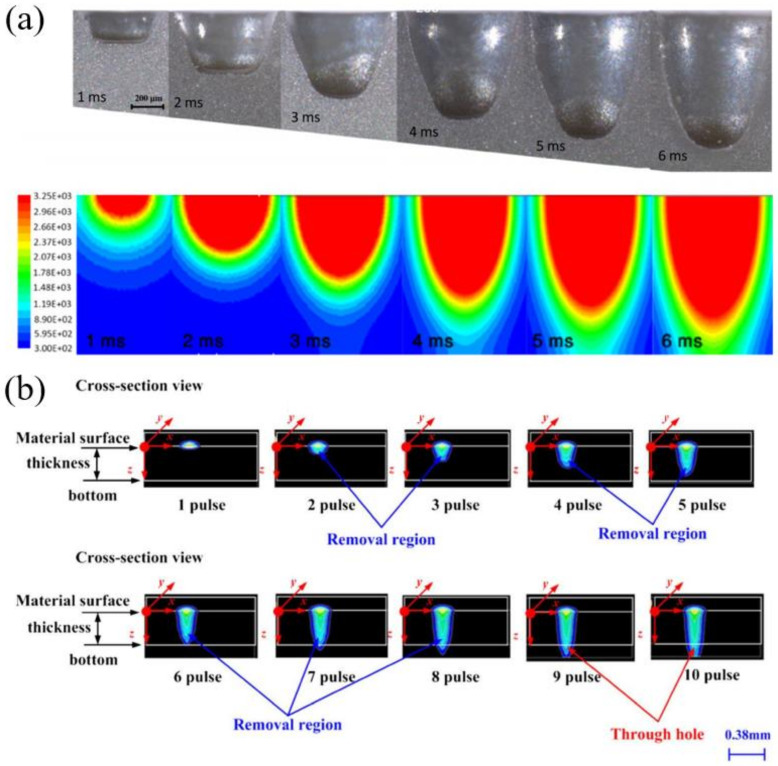
(**a**) The hole drilled by the experiment and the corresponding simulation results. Reprinted with permission from ref. [31]. Copyright 2012 Elsevier; and (**b**) the simulation results of the drilling process under different laser pulses. Reprinted with permission from ref. [32]. Copyright 2015 Springer.

Then, a two-dimension axisymmetric finite element model was developed to simulate the temperature and precede hole formation during the drilling [31,32]. As shown in Figure 5a, the simulated temperature distributions showed that the vaporized area and the actual ablated hole maintained high similarity. Additionally, Chen et al. reported similar simulation results in 2015, as shown in Figure 5b [32].

### 3.2. Percussion Drilling

**Figure 6:**
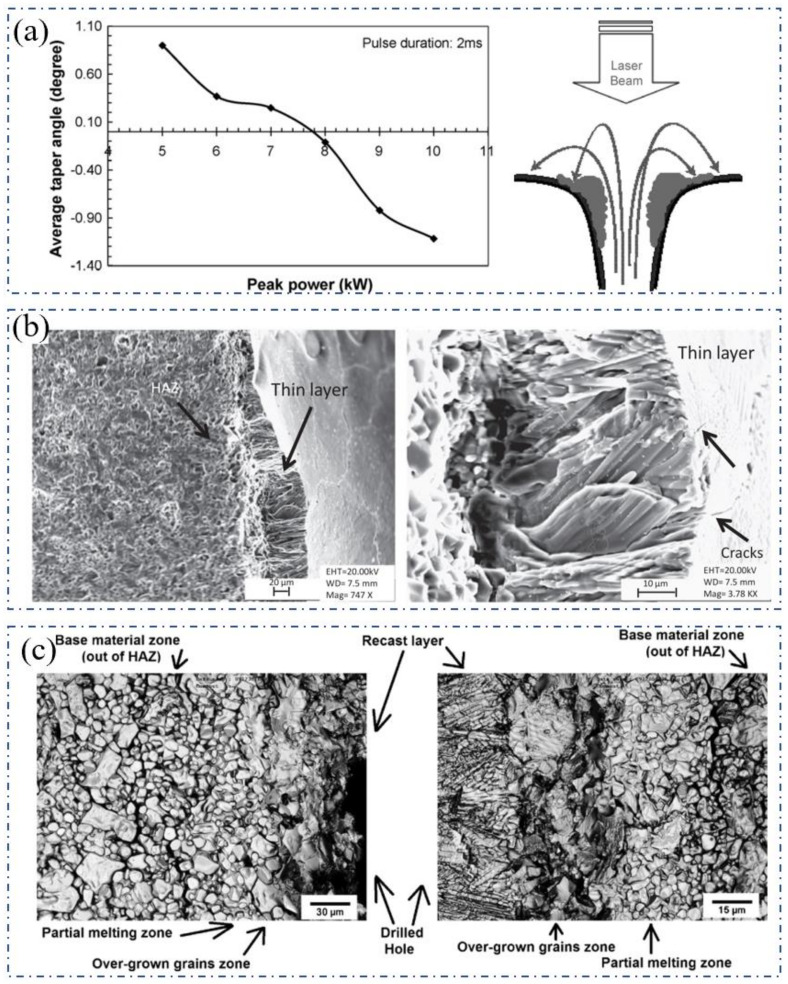
(**a**) Hole taper angle under the drilling of the laser with different peak power. Reprinted with permission from ref. [33]. Copyright 2009 Elsevier; (**b**,**c**) and the cross-section of the hole wall. Reprinted with permission from ref. [34]. Copyright 2013 Elsevier.

In the millisecond laser percussion drilling of ceramics, finding the optimal drilling parameters is the long-term pursuit of researchers. In 2009, Kacar et al. reported the experimental results of the laser drilling of alumina ceramic with a thickness of 10 mm. The wavelength was 1064 nm, the focus diameter was 480 μm, the pulse duration was 0.5–8 ms, and the peak power was 0.5–11.0 kW. The results showed that when the millisecond pulse peak power was fixed, the diameter of the hole lower surface was proportional to the pulse duration, but there was no such change trend in the diameter of the hole’s upper surface. At the same time, when the pulse duration was fixed, the diameter of the hole lower surface was proportional to the peak power of the laser pulse. Additionally, there was no such change trend in the diameter of the upper surface. Therefore, holes with a negative taper could be drilled, as shown in Figure 6a. It was suggested that the re-solidification of the molten material decreased the diameter of the upper surface of the hole. When the laser pulse duration was longer or the peak power was higher, more vaporized material would re-solidify at the entrance position so that the diameter of the upper surface was reduced, while the diameter of the lower surface continued to increase. Therefore, the taper of the hole could be effectively controlled [33].

Hanon et al. further studied the millisecond pulsed laser drilling of alumina ceramics with different thicknesses (5 and 10.5 mm) from theoretical and experimental aspects. The peak power (5–9 kW), pulse duration (1–6 ms), pulse number (10–100), repetition rate (5–20 Hz), and negative defocus (0–4 mm) on the drilling performance were systematically studied. In general, the increase in the peak power, pulse duration, and pulse number would initially increase the diameter of the holes, and gradually showed a trend of saturation, and a negative defocus would increase the hole taper angle (the effect of positive defocus was not studied) [31]. This regular patten was also proved by the study of Yan et al. which used a 3.5 kW CO_2_ millisecond pulsed laser [34]. In addition, the authors found that there were remelted layers with a thickness of 10–50 μm in the inner wall of the hole. From the microscopic SEM picture in Figure 6b, the remelted layer was columnar grains that faced toward the hole wall, and these columnar structures caused cracks under the action of the stress generated by the laser heating and cooling. The microstructure of the inner wall of the hole was further confirmed by Yan et al. in 2012, as shown in Figure 6c [34]. The upper surface of the hole was divided into three parts: including the recast layer, over-grown grains zone, and partial melting zone. From the cross-sectional view of the hole wall, it can be seen that the crystal growth direction was also facing the hole wall (along the temperature gradient direction). Among them, the recast layer was caused by the molten alumina that was not eliminated during the drilling process; the over-grown grains zone was caused by the further growth of the crystals due to the significant heat effect during laser drilling. The partial melting zone was caused by the energy transferred through thermal conduction.

**Figure 7:**
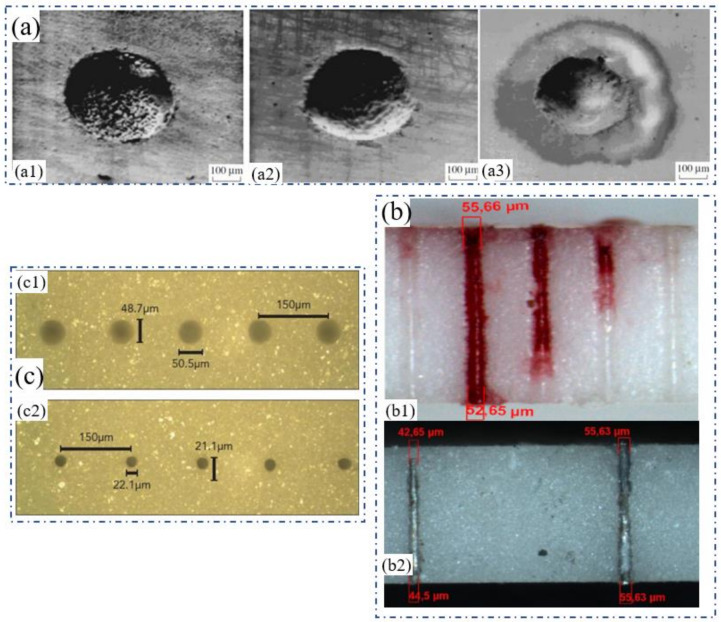
(**a**) The blind hole drilled by the microsecond laser with (**a1**) 0.15 μs, 61 J/cm^2^, 200 pulses, (**a2**) 1.1 μs, 110 J/cm^2^, 50 pulses, and (**a3**) 4.5 μs, 91 J/cm^2^, 20 pulses. Reprinted with permission from ref. [35]. Copyright 2004 IOP; (**b**) the cross-section of holes drilled in (**b1**) Al_2_O_3_ and (**b2**) AlN. Reprinted with permission from ref. [36]. Copyright 2015 Elsevier; and (**c**) the hole upper surface (**c1**) and lower surface (**c2**) drilled by the QCW laser. Reprinted with permission from ref. [37]. Copyright 2015 AIP.

The technological maturity of the laser system provides new technical means for ceramic drilling. Based upon this, researchers have further explored the high-efficiency and high-quality micro-hole processing under the new parameters, making the laser drilling of ceramic materials gradually close to the market. In 2004, Garnov et al. obtained a microsecond pulsed laser output with a pulse energy of up to 100 mJ through a specific laser system. The pulse duration of 1.1 and 4.5 μs had a maximum pulse energy of 400 mJ, the pulse duration of 0.15 μs had a maximum pulse energy of 200 mJ, and the repetition rate of the microsecond laser was 1–10 Hz. As shown in Figure 7a, when the pulse duration was 0.5 and 1.1 μs, sharp crater edges and a small amount of sputtering deposition is visible near the edges. For the ablation of the 4.5 μs laser, there was obvious thermal damage outside the crater, which could not be explained by heat diffusion from the irradiation spot which was probably caused by the plasma formed in the vapor plume. Therefore, for drilling alumina ceramic, a pulse duration of 1.1 μs seems to be a compromise between drilling efficiency and drilling quality [35].

In recent years, with the rapid development of fiber lasers, CW fiber lasers and quasi continuous (QCW) fiber lasers have shown great advantages in ceramic drilling [38]. In 2015, Adelmann and Hellman reported the results of single-mode fiber lasers in the high-speed drilling of ceramic substrates. The experiment used a 1070 nm, 500 W single-mode CW laser, which could output a pulsed laser with then shortest pulse duration of 20 μs through modulation. The focal length of the focusing lens was 50 mm, the laser Rayleigh length was calculated to be 70 μm, and the focal spot diameter was 11 μm. The auxiliary gas nitrogen was coaxially output with the laser beam with a purity higher than 99.99% (the diameter of the gas nozzle was 0.3 mm and the pressure was between 6 and 14 bar). The thickness of the alumina ceramic sample was 0.635 mm and the laser absorber was used for enhancing the drilling performance. Experiments showed that when the laser was focused on the sample surface, a hole with a diameter of 55.66 μm on the upper surface and a diameter of 52.65 μm on the lower surface was obtained, as shown in Figure 7(b1). The standard deviation of the diameter of 20 holes was only 3.5 μm, which showed drilling with high efficiency, quality, and stability. Additionally, the AlN substrate could also be well drilled (Figure 7(b2)) [36]. This research shows the obvious advantages of single-mode CW laser in the high-speed drilling of ceramics. However, the disadvantage is the dependence on the laser absorber and high-pressure nitrogen assistance.

In fact, the ceramic drilling of the QCW laser was reported by Mendes in 2014 with an excellent processing result. Figure 7c shows holes drilled on 96% Al_2_O_3_ with a thickness of 635 μm (300 holes/s, a pitch of 150 μm, and a linear moving speed of 45 mm/s). The exit of the holes was manually measured under optical microscope to be 22 ± 3 μm and the entrance was 49 ± 3 μm. In addition, it was demonstrated that 100 μm alumina at a rate of 3000 holes/s could be achieved by the QCW laser [37]. In general, the application of the single-mode CW fiber laser and QCW fiber laser in ceramic drilling has greatly broken through the limitations of the diameter and drilling speed of a conventional solid-state laser. At present, it has also become the most popular choice in industrial applications.

### 3.3. Trepan Drilling

**Figure 8:**
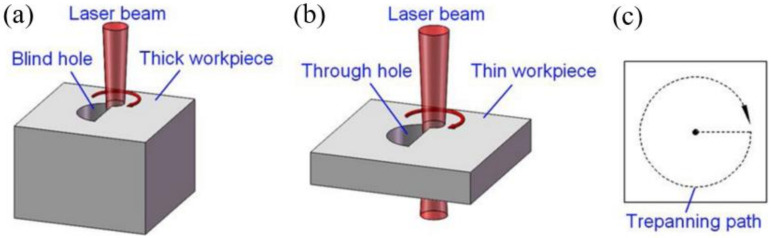
Schematics of the laser trepan drilling of a (**a**) blind hole; (**b**) through hole; and (**c**) the trepan path. Reprinted with permission from ref. [42]. Copyright 2019 Springer.

Laser trepan drilling, which can be used to drill meso-to macro-sized holes in structural ceramics, has become the most widely used drilling method [39,40,41]. During trepan drilling, a high-intensity laser is focused on the target surface that can melt and/or vaporize the target, and pressurized auxiliary gas jets are often used to eject molten material from the processing area. The laser beam removes the work material along the periphery of the hole to be drilled. Figure 8 shows the schematics for illustrating the laser trepan drilling method [42].

**Figure 9:**
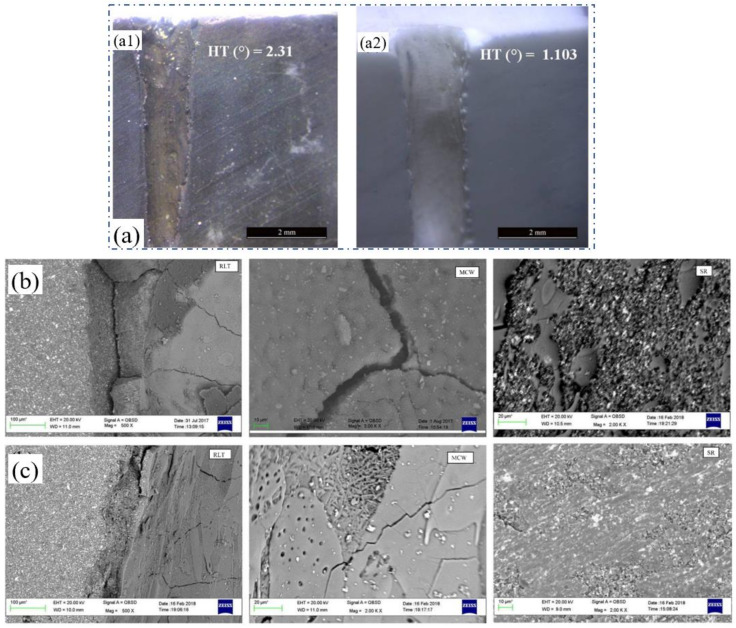
(**a**) Cross-section of the hole drilled by (**a1**) non-optimal parameters and (**a2**) optimal parameters (4 ms, 8 Hz, 5 mm/min, and 9 kg/cm^2^). Reprinted with permission from ref. [43]. Copyright 2018 Elsevier. SEM images of the cross-section of the hole drilled in ZTA at (**b**) worst and (**c**) optimum levels). Reprinted with permission from ref. [41]. Copyright 2018 Elsevier.

In 2018, Saini et al. studied the effect of the process parameters on the hole characteristics such as hole circularity, hole taper, and spatter size during the millisecond laser trepan drilling of the ZTA. A 1064 nm, 10 kW peak power, 400 μm millisecond pulsed laser was used for drilling ZTA ceramic with a thickness of 6.0 mm. It was suggested that low pulse duration was recommended to obtain a better hole geometry and higher gas pressure would increase the spatter size. Figure 9a shows the comparison of the hole quality between the non-optimal and optimal parameters. It is obvious that the hole taper and spatter size were reduced [43]. Then, in 2019, Saini et al. further studied the effect of process parameters on the hole recast layer thickness, crack width, and surface roughness by using a genetic algorithm to determine the optimal processing parameters. Multi-objective optimization results suggest an overall improvement of 14.93% (4 ms, 10 Hz, 20 mm/min, and 9.5 kg/cm^2^) in multiple quality characteristics with individual improvements in recast layer thickness of 7.25%, surface roughness of 27.16%, and crack width of 10.38% when compared with the average experimental values. As shown in Figure 9b,c, the SEM images of laser-trepanned holes at predicted optimum parameter values justify these improvements [17].

**Figure 10:**
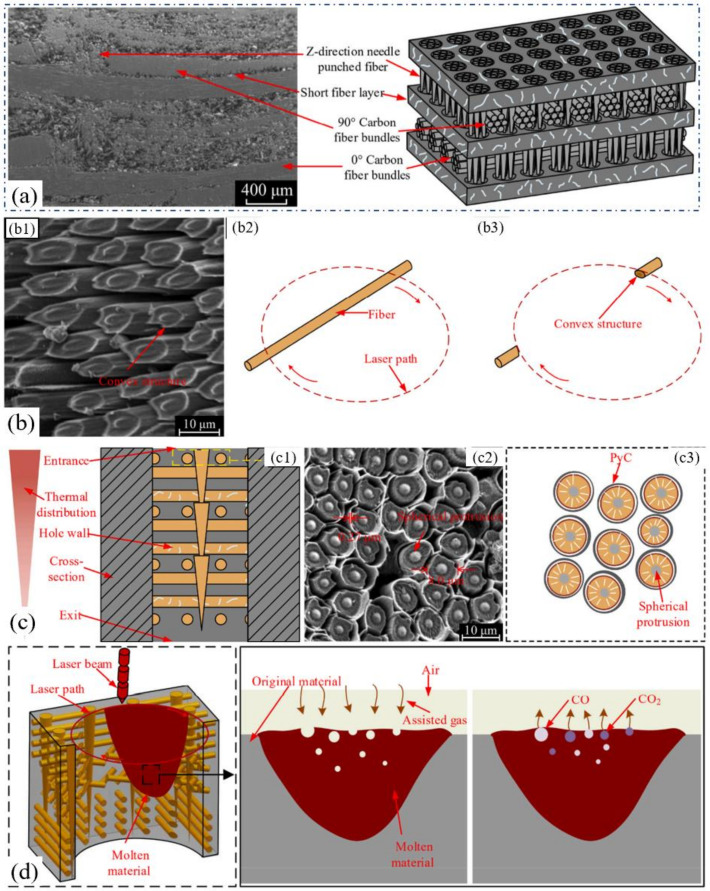
(**a**) Cross-section of the 2.5D C_f_/SiC composites; (**b**) convex structure obtained by the laser ablation of the 0° fiber, (**b1**) represents the schematic diagram of the fiber before laser drilling, (**b2**) represents the schematic diagram of the fiber after laser drilling, b3 represents the enlarge of different ablation topographies; (**c**) spherical protrusion obtained by the laser ablation of the 90° fiber, (**c1**) represents the selected observation positions on the hole wall, (**c2**) represents the schematic diagram of the formation of the ablation topography, (**c3**) represents the observed evolution of the ablation topographies of the fiber during the drilling; and (**d**) the formation of the bubble particle. Reprinted with permission from ref. [19]. Copyright 2021 Elsevier.

The above ceramic materials have a stable internal structure, and the removal process under the irradiation of millisecond laser shows repeatability. However, the 2.5D C_f_/SiC composite that has a special structure characteristic, as shown in Figure 10a, was a key heat-resistant ceramic matrix composite in an aerospace field [19,44]. Recently, Liu et al. reported the drilling results of the 2.5D C_f_/SiC composites. A QCW fiber laser (IPG YLR-150/1500) with a pulse duration of 0.4 ms was used to drill the target under the trepan method (1070 nm, 25 μm). Figure 10a shows the schematic diagram of the 2.5D C_f_/SiC composites, and one of the ablation topographies is shown in Figure 10b. The 0° fiber experienced ablation evolution from a filiform connection, flat ellipse to needle-like form to the convex structure during the drilling. Correspondingly, the 90° fiber suffered the ablation evolution from the cylindrical structure, lotus lead shape, and bud-shape to closed shape. The core of the carbon fibers experienced the topography structure from cylindrical protrusion (as shown in Figure 10c), spherical protrusion to a needle-like form. Under the irradiation of the laser energy, the C_f_/SiC was transformed into a molten state, and then into boiled molten liquid. When the induced CO and CO_2_ left the inside of the material, bubble particles eventually formed, as shown in Figure 10d [19].

In addition, the key laser parameters such as scanning speed, pulse duration, repetition rate, defocus amount, and pulse energy were selected to study their interaction effects on the hole taper and HAZ. The damage characteristics and ablation mechanism were also studied. The results showed that the fiber laser trepan drilling integrated with the proposed experimental and optimization analysis achieved the high-quality micro-hole machining of the 2.5D C_f_/SiC composite [19,44,45].

**Figure 11:**
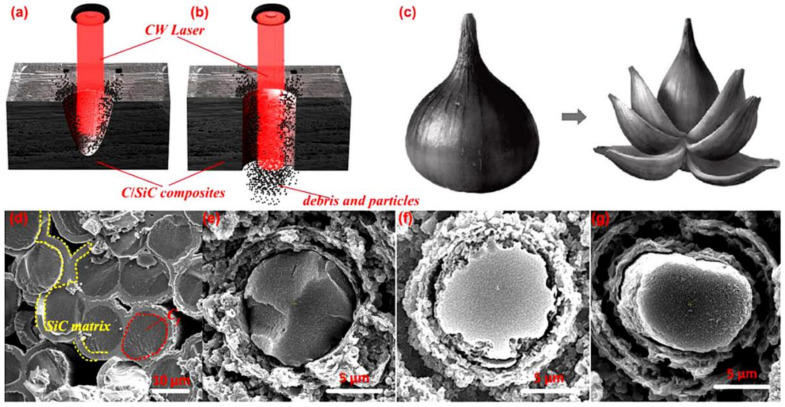
(**a**,**b**) The two different laser machining processes; (**c**) schematic diagram of striping onions; and (**d**–**g**) ablation morphology at different ablation stages. Reprinted with permission from ref. [46]. Copyright 2021 Elsevier.

Moreover, in 2021, Zhang et al. applied a CW laser to the trepan drilling of a C_f_/SiC ceramic composite with a thickness of 3 mm. The authors pointed that CW lasers could perform the steady, cost-effective, and precise processing of C_f_/SiC composites. A CW laser (1067 nm, 100 Hz, 80 μs, 30–80 W) was used to drill holes with diameters of less than 1 mm, and the effects of the laser parameters on hole quality were analyzed. It was proven that the CW laser drilling was highly efficient and cost-effective. As shown in Figure 11a,b, when the hole depth was less than the target thickness, the induced shock wave would create a recoil pressure and eject some debris and particles. Then, a through hole was formed as the drilling time increased, and most debris and particles would flow through the hole lower surface. In addition, the ablation of the SiC matrix occurred earlier than that of carbon fiber since the latent heat of the carbon fiber was nearly three times larger than that of SiC. Therefore, after the outer layers were first ablated, the SiC matrix would be ablated layer by layer, as shown in Figure 11e–g, similarly to the process of stripping an onion (Figure 11c) [46].

## 4. Short Pulsed Laser Drilling of Ceramic

### 4.1. Drilling Mechanism

The drilling mechanism of the nanosecond laser is similar to that of the millisecond laser. The material can be removed through evaporation, dissociation and ejection in the melt. However, the nanosecond laser usually has a peak power density higher than 10^9^ W/cm^2^. It can easily ablate the material and remove it, which improves the vaporization ratio of the material removal. In contrast to the melt sputtering under the irradiation of the millisecond laser, the thermal effect of the nanosecond laser drilling will be significantly reduced. On the other hand, the high fluence of the nanosecond laser will create a plasma plume. The shielding effect of the plasma should be carefully considered when analyzing the drilling of the nanosecond laser from a theoretical point of view, which has been proven by Atanasov in 2001 [47] and Ho and Lu in 2003 [48].

### 4.2. Percussion Drilling

The limited pulse energy and the plasma shielding of the nanosecond laser make percussion drilling difficult. Therefore, it is more important to find laser parameters suitable for percussion drilling. Biswas et al. summarized the influence of the different processing parameters, such as pulse energy, repetition rate, pulse duration, air pressure and focal length on the drilling performance [21,49,50]. However, even if the drilling works under the optimal parameters in the above research, the drilling performance is still limited to meeting the current demand for drilling [51]. To improve the drilling quality, in 2021, Shaheen et al. used a 193 nm excimer laser (4 ns, 1–300 Hz, 3–160 µm) for the percussion drilling of Al_2_O_3_ with a thickness of 0.5 mm. The effect of the laser parameters on the drilling efficiency and drilling quality was investigated. It was proved that higher ablation efficiency could be produced using a smaller laser spot, and that minimal thermal effects and the absence of cracks could be achieved [52].

**Figure 12:**
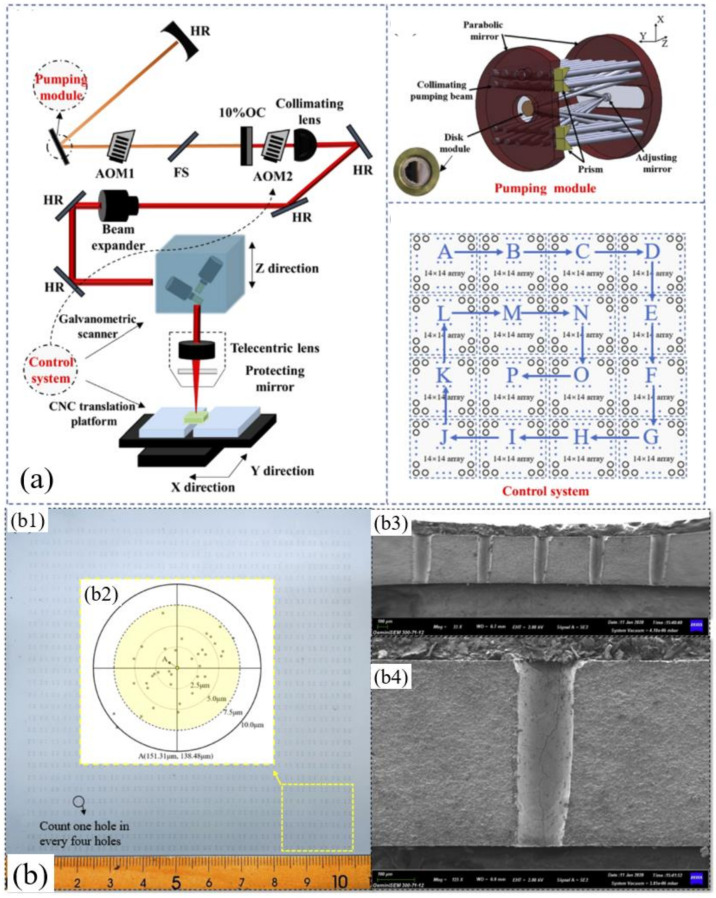
(**a**) The experimental system, pump module and the control system; (**b1**) the drilled hole arrays; (**b2**) the distribution of the position error of holes; (**b3**,**b4**) the cross-section of one hole [53].

In our previous work, to avoid the influence of the plasma shielding, we suggested that a sub-microsecond laser could be used as a potential laser system for the high-speed drilling of ceramics. An appropriate pulse duration (>500 ns), a high pulse energy (>5 mJ), and a medium peak power (<30 kW) were able to obtain a higher MRR. Then, as shown in Figure 12a, a sub-microsecond laser (1030 nm, 611.6 ns, 20 Hz, 7.5 mJ, 50.57 µm) was introduced for the high-speed drilling of the 96% Al_2_O_3_ with a thickness of 0.5 mm. The cyclic scanning path was used to avoid stress concentration in drilling, so as to avoid the chipping phenomenon of the ceramic substrate. Additionally, by adopting positive defocusing (1.3 mm), holes with a diameter difference of ±10 µm were obtained. Figure 12b shows the drilling results of 3136 holes, with a total time of 104 s; thus, a speed of ~30 holes/s was achieved. Additionally, a high drilling quality was also shown (2–4 µm recast layer, 10 µm HAZ, fewer median cracks and lateral cracks), as shown in Figure 12. The performance of the sub-microsecond pulsed thin disk laser provides a reference in the field of high-speed laser processing [53].

**Figure 13:**
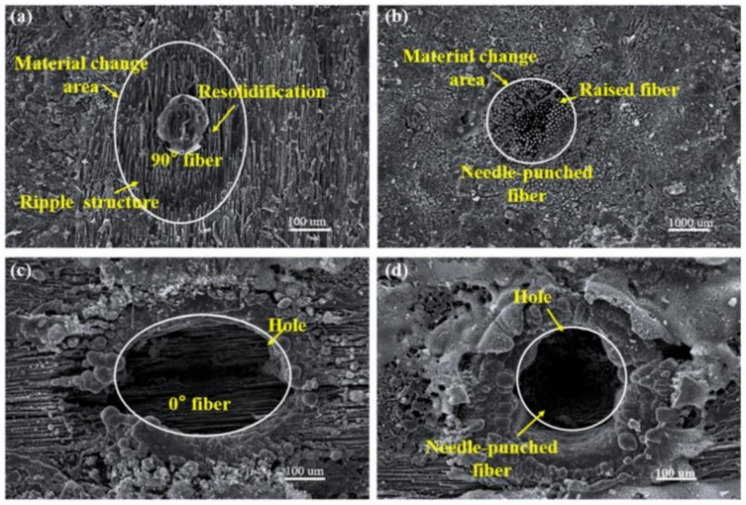
The morphology of the ablation: (**a**) 90° fiber direction (10 W, 10 s); (**b**) *Z* axis needle-punched fiber direction (10 W, 10 s); (**c**) 0° fiber direction (39 W, 5 s); and (**d**) *Z* axis needle-punched fiber direction (39 W, 5 s). Reprinted with permission from ref. [54]. Copyright 2021 Springer.

In addition, the nanosecond laser percussion drilling of C_f_/SiC composites was also recently reported. In 2021, Jiao et al. used the nanosecond laser (20 ns, 200 kHz, 28 µm) for the percussion drilling of 2.5D C_f_/SiC composites. As shown in Figure 13a, an ellipse shape of the re-solidification was shown in the 90° fiber under the irradiation of a low laser fluence (an average power of 10 W). The *Z* axis needle-punched fiber was also not removed under the same laser fluence, as shown in Figure 13b. Under the irradiation of a middle laser fluence (39 W), a blind hole which was an oval hole was observed at the surface of the 0° fiber, as shown in Figure 13c, and the blind hole with a circular shape in the needle-punched fiber area was smaller and deeper [54]. The experimental results confirmed that, for this kind of composite structural ceramic, there will be holes with different morphologies ablated under the irradiation of the same laser fluence since the different structures of the ceramic have different laser absorption, which is consistent with the conclusions of Liu et al. [19,44].

### 4.3. Trepan Drilling

**Figure 14:**
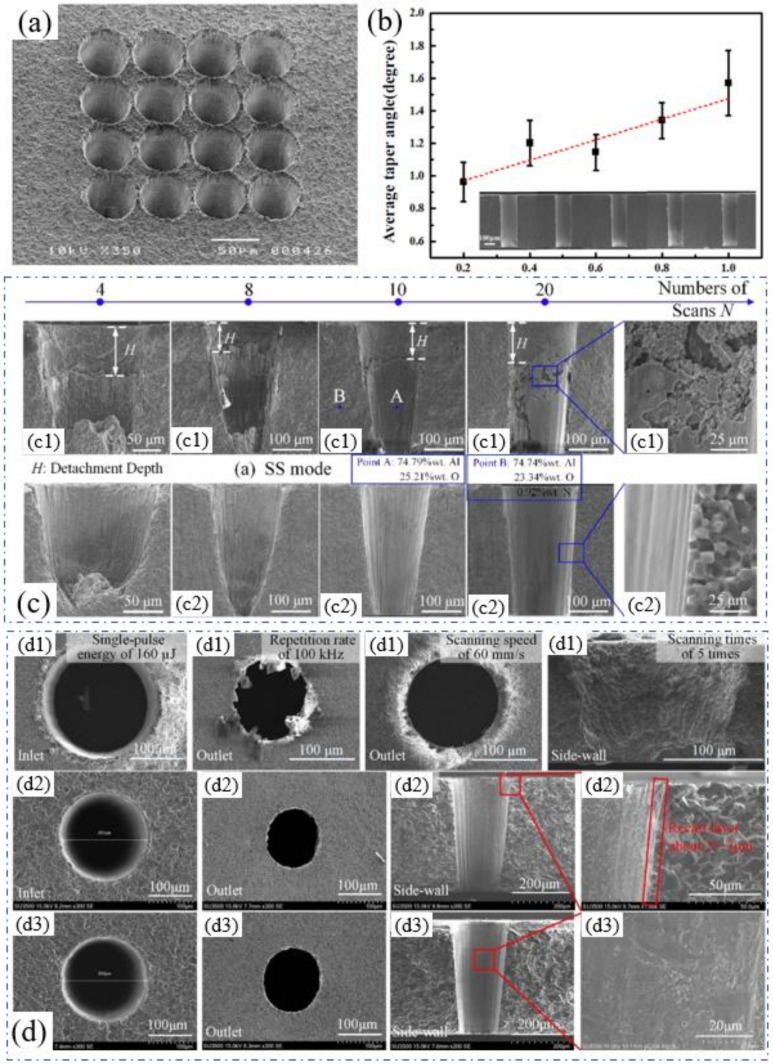
(**a**) A 40 μm hole array (250 μm thickness, 60 μm pitch). Reprinted with permission from ref. [55]. Copyright 2007 Springer; (**b**) the relationship between the average taper angles and the scanning pitch. Reprinted with permission from ref. [56]. Copyright 2018 Elsevier. The morphology of the hole wall drilled by (**c1**) outside/in (**c2**) inside/out jump direction. Reprinted with permission from ref. [57]. Copyright 2020 Elsevier; the hole drilled by the (**d1**) non-optimal laser parameters, (**d2**) optimal laser parameters by filled spiral laser trepan, and (**d3**) optimal laser machining parameters by multiple rings laser trepan [11].

In 2007, Knowles et al. showed the results of high-quality hole arrays, as shown in Figure 14a, which was drilled using a 20 ns copper vapor laser (511 and 578 nm, 3 W, 10 kHz, 20 ns, 6 s/hole) and demonstrated the ability to drill high-density hole arrays in ceramics without cracks. It was suggested that although ceramics are prone to cracks due to thermal stresses, this could be avoided with the optimized processing strategies maintaining a low heat input to the bulk material [55]. Then, in 2018, Wang et al. used a 532 nm laser (0.86 J/mm^2^ laser fluence, 100 kHz repetition rate, 150 µm hole diameter, 0.2–1 µm pitch,200 mm/s scanning speed, and scanning 20 times for a single hole) for the trepan drilling of Si_3_N_4_. Figure 14b shows the relationship between the average taper angles and the scanning pitch. The results show that as the scanning pitch increased, the average taper angle increased, and a cylindrical hole with a minimum taper angle of ~0.96° was obtained at a scanning pitch of 0.2 µm. In addition, this also shows that the surface roughness of the hole cross-section increased with the increase in scanning pitch, which might be due to the change in laser energy accumulation at different scanning pitches, and the number of debris changed due to the change in ablation threshold with the different surface roughnesses [56].

In addition to the significant influence of scanning speed on the hole quality, previous have shown that the scanning direction and scanning path also have an effect. In 2020, Wang et al. studied the hole trepan on AlN using a nanosecond pulsed laser. A 355 nm pulsed laser with 10–23 ns, 40–150 kHz, 22.5 µm and 10 W was adopted for the trepan drilling of 96% AlN ceramics with a thickness of 0.38 mm. It was proven that the actual diameter of the drilled hole was the combination of the scanning path setting and actual drilling. Figure 14c shows the cross-section of holes on AlN with two different jump directions: outside/in and inside/out. The study found that the inside/out drilling method produced better hole quality, in which the hole wall was smoother, but presented vertical stripes [57].

Then, in 2021, Zhao et al. further analyzed the influence of laser trepan patterns on drilling performance. A 355 nm pulsed laser with 11 ns, 50 kHz, 20 µm and 10 W was adopted for the trepan drilling of 96% Al_2_O_3_ samples with a thickness of 0.12–0.50 mm. Two laser trepan patterns were evaluated, which filled spiral trepan and multiple rings trepan. The hole drilled by the non-optimal laser parameters is shown as Figure 14(d1), displaying lots of burrs, spatters, and a high HAZ, etc. For comparison, the holes drilled with the optimized laser parameters are shown in Figure 14(d2,d3). The optimized laser machining parameters are as follows: 120 µJ, 50 kHz, 4000 mm/s laser jump speed, 300 mm/s scanning speed, and 300 scanning times. In addition, the filling pitch of the filled spiral laser trepan and multiple rings laser trepan was 15 and 10 µm, respectively. It is evident that no micro-cracks, spatters, and HAZ appeared on the hole wall, and a thin recast layer with a thickness of 2–3 µm was obtained, while the lower surface of the hole showed an elliptical shape and a small number of burrs. In other words, issues such as the cracks, recast layer, HAZ, and spatters, could be practically eliminated by using the optimized laser parameters [11].

## 5. Ultrafast Pulsed Laser Drilling of Ceramic

### 5.1. Drilling Mechanism

**Figure 15:**
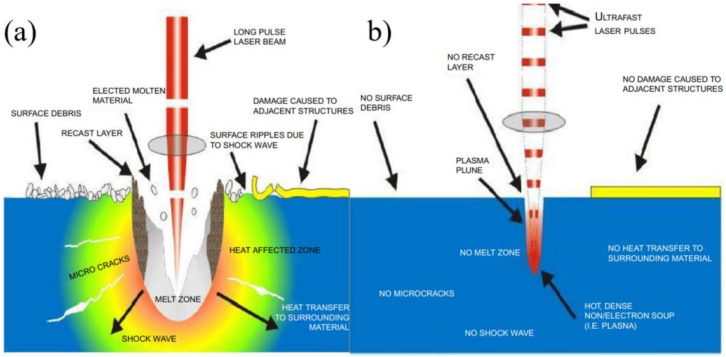
Schematic effect of the laser–matter interaction during (**a**) long (or short) pulsed laser and (**b**) ultrafast pulsed laser [60].

The ultrafast laser has become an alternative tool for ceramics processing due to its ultra-high laser intensity and ultra-short laser pulses. The ultra-high laser intensity enables the ultrafast laser to ablate any target materials, and the ultra-short laser pulse helps deliver the laser energy into the target before thermal diffusion occurs [14,58,59]. In addition, more precise processing features with minimal HAZ could be achieved. At present, the ultrafast laser was proven to be an efficient tool for the drilling of structural ceramics, such as SiC, Al_2_O_3_, ZnO and composite ceramics. Compared with the long or short pulsed laser, the amount of molten material and the zone affected by residual heat are significantly reduced due to shorter pulse duration, as shown in Figure 15. Therefore, compared with the millisecond or nanosecond laser drilling analyzed above, the ultrafast laser (picosecond laser and femtosecond laser) can provide a treatment of better quality and efficiency, especially for the drilling of hard and brittle ceramic materials.

In 1995, Ihlemann studied the UV laser ablation behavior of various ceramics (Al_2_O_3_, MgO and ZrO_2_) using the lasers with different wavelengths (248 nm, 308 nm) and pulse durations (30 ns, 500 fs). It was shown that, using sub-picosecond pulses, the ablation threshold was generally lower than that of the nanosecond laser. In the process of nanosecond laser drilling, the plasma-mediated ablation dominated, while in the femtosecond case, the process controlled by multi-photon absorption was able to micro-structure the material. The processing of the femtosecond laser enabled ablation without thermal influence, so that structuring with µm-resolution could be achieved [61].

### 5.2. Percussion Drilling

Since the ablation of the ultrafast laser creates a high-pressure shock wave, the cracks may be generated in the ceramic by the tensile. Therefore, it is difficult to perform precise processing because of the significant damage formed around the processed region. Usually, in order to ensure the quality of ultrafast laser drilling, the laser fluence of the laser will be set near the ablation threshold of the material, so as to avoid damage in the drilling as much as possible, which makes it difficult to process high-aspect-ratio holes through percussion drilling (it can be a disadvantage of percussion drilling, since the cycle for producing holes with a given size cannot be sped up by raising the laser power).

**Figure 16:**
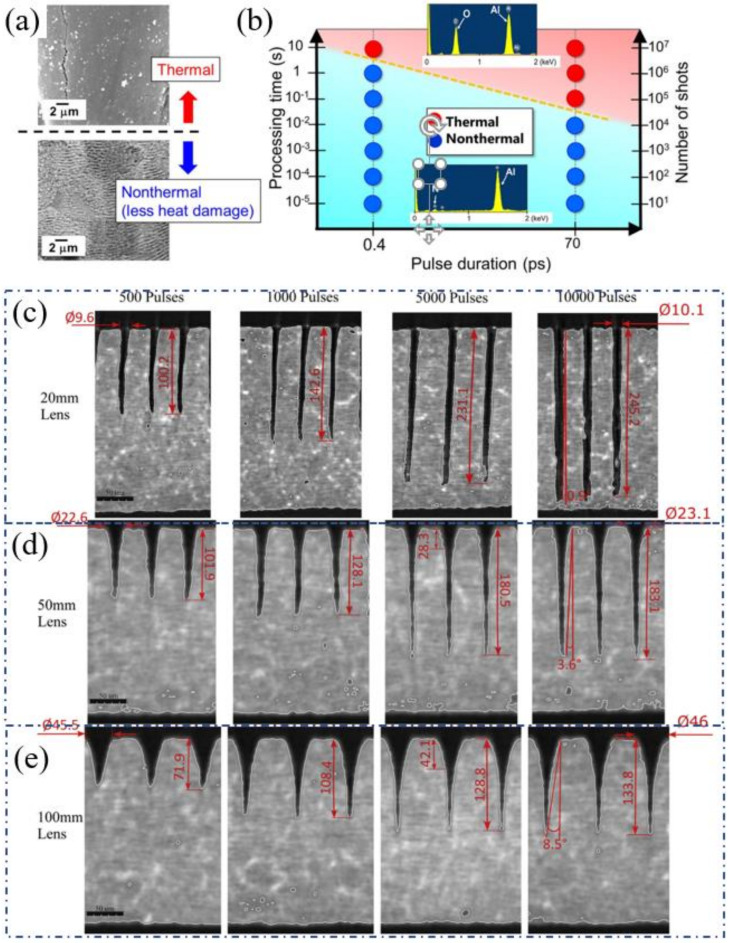
(**a**) AlN ceramic drilled by laser percussion method; (**b**) the influence of the pulse duration and pulse number on the drilling morphology. Red circles represented the upper figure in (**a**), and blue circles represented the lower figure in (**a**). Reprinted with permission from ref. [62]. Copyright 2020 Springer; and (**c**–**e**) the cross-section drilled under three lenses (20 mm, 50 mm, 100 mm) by irradiating 500, 1000, 5000 and 10,000 pulses. Reprinted with permission from ref. [63]. Copyright 2018 Elsevier.

In 2020, Narazaki et al. studied the nonthermal–thermal processing boundary in the drilling of ceramics (AlN and YSZ) using an ultrashort pulsed laser system with a pulse duration which varied from 400 fs to 400 ps. It was found that AlN ceramic exhibited a strong dependence of the ablation rate on the laser fluence. As shown in Figure 16a, there was a nonthermal–thermal process boundary, and the boundary depended on the pulse duration (as shown in Figure 16b). In addition, the ablation behavior of YSZ was different from that of AlN since the processing of YSZ has no nonthermal process window. Therefore, the boundary of the nonthermal–thermal process was determined by the laser parameters and the thermal conductivity of the material [62].

To improve the aspect ratio of the hole drilled by the ultrafast laser, Nasrollahi et al. studied the influence of the lenses with different focal lengths on the holes’ aspect ratio. A 1030 nm, 310 fs, 500 kHz, 5 W femtosecond laser was used to percussion drill the Si_3_N_4_ with a thickness of 0.25 mm. The focusing lenses with a focal distance of 100, 50 and 20 mm were used to obtain a beam diameter of 45, 23 and 9 µm, respectively. The depth of focus was calculated to be 2375, 621 and 95 µm, respectively. The results are shown in Figure 16c–e. It was proven that the aspect ratio of the hole could be increased from 3 to 25 by changing the focal length of the lens, and the highest aspect ratio was obtained with a focal length of 20 mm. In addition, it was also demonstrated that increasing the laser fluence and/or decreasing the focal length improved the drilling quality, namely in terms of the taper angle and circularity of the hole. Therefore, it was proven that a small focal distance lens was helpful for the percussion drilling of micro-holes [63]. Then, in 2020, a beam shaping system for laser percussion drilling was designed and implemented to obtain holes with a top-hat spatial profile. The results show that the laser with Gaussian beam could obtain a higher penetration depth and a higher aspect ratio, while the use of a top-hat beam could improve the geometric accuracy of the hole. In addition, according to the ablation threshold, the spatial distribution of the top-hat beam could be precisely adjusted to minimize the HAZ and injected laser fluence [12].

**Figure 17:**
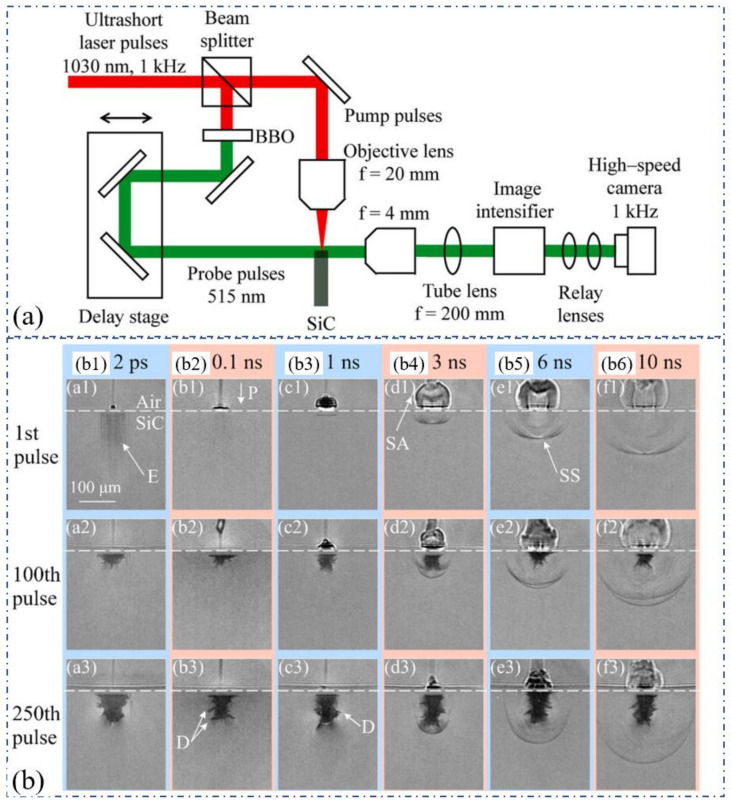
(**a**) Optical setup for experiments. Shadowgraphs at (**b1**) 2 ps, (**b2**) 0.1 ns, (**b3**) 1 ns, (**b4**) 3 ns, (**b5**) 6 ns, (**b6**) 10 ns after (1) 1st, (2) 100th, and 250th. Reprinted with permission from ref. [64]. Copyright 2021 Elsevier.

Recently, in order to study the damage process caused by the stress waves in the femtosecond laser drilling process, a pump probe imaging system with high temporal resolution was used by Hattori et al. to study the transient shock wave changes and high-speed dynamic process of crack formation in the process of femtosecond laser drilling. A 1030 nm, 180 fs, 1 kHz, 200 µJ, 5.7 µm laser was used as the pump pulse for the percussion drilling of the SiC with a thickness of 0.35 mm. The probe pulse was a 515 nm femtosecond laser obtained through the 1030 nm femtosecond laser under the frequency doubling of the BaB_2_O_4_ (BBO) crystal, which passed through the target from the direction perpendicular to the pump pulse after delaying the process using an optical delay stage. On the other hand, a probe pulse was focused by an objective lens with a focal length of 20 mm. Figure 17b shows the hole shape and femtosecond laser-induced high shock which were observed pulse to pulse. It was apparent that the propagation speed of the pressure waves in the air were much higher than that in SiC as observed at 1 ns after the irradiation of the first ssecond laser pulse, as shown in Figure 17(b1). However, the propagation distances of the pressure waves in the air and SiC in the axial direction became almost equal at 6 ns, whereas at 10 ns, the propagation distance of the pressure wave in SiC exceeded that in the air. In addition, the damage observed after the irradiations of 250 pulses (surrounded by yellow circles) did not exhibit a shape change even after more pulses were irradiated. These characteristics and the fact that the stress waves were generated at the tip of the hole suggest that the propagation of stress waves resulted in fracture; consequently, cracks appeared and were observed as damage [64].

In addition, since the microrough implants exhibited a better osseointegration than smooth ones, the femtosecond laser processing method was successfully implemented to produce micro-grooved implants which performed well both in vitro and in vivo. In this context, A. Stanciuc et al. reported a new application of femtosecond laser drilling ceramics. A 795 nm, 120 fs, 1 kHz femtosecond laser was used to drill multi-blind hole with a diameter range from 10 to 30 µm in the ZrO_2_ surface, which showed high precision and reproducibility. The results showed that the patten 30 um diameter/10 um depth induced the strongest osteoblastic human mesenchymal stem cell commitment. This study confirmed the possibility of the wide application of the femtosecond laser drilling of bio-ceramics in the medical field [65].

### 5.3. Trepan or Helical Drilling

**Figure 18:**
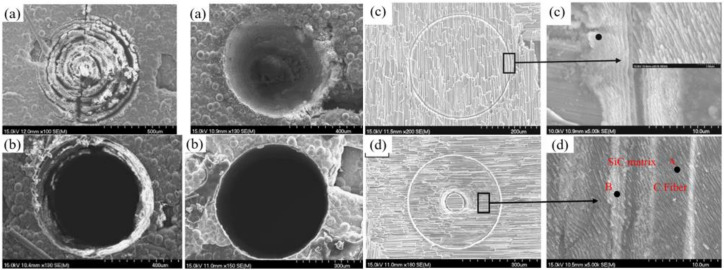
(**a**) A donut shape drilled with helical lines spacing at 0.2 (**left**) and 0.05 mm (**right**); (**b**) the upper surface (**left**) and lower surface (**right**) of the through hole. Reprinted with permission from ref. [66]. Copyright 2012 Springer. SEM images of C_f_/SiC after (**c**) single ring line drilling and (**d**) helical lines scanning with 0.01 J/cm^2^. Reprinted with permission from ref. [67]. Copyright 2013 Springer.

For the research on picosecond laser drilling in ceramic materials, in 2013, Wang et al. used a 1 ps (1030 nm, 100 kHz) laser to drill a blind hole and used a 10 ps (532 nm, 20 W) to drill a through hole in C_f_/SiC with a thickness of 3 mm. A donut shape could be drilled by the 1 ps laser with helical lines spacing at 0.2 mm and a scanning speed of 1000 mm/s, and a blind taper hole was obtained by adjusting the helical lines spacing to 0.05 mm, as shown in Figure 18a,b. In addition, it was found that the hole depth decreased with the increase in the laser scanning speed, but the diameter did not change much. In order to obtain a good quality through hole, the hole was drilled under a 10 ps laser three times: in the first step, the parameters of 19.5 W and 400 kHz were adopted; the second step adopted 17.5 W, 400 kHz, and finally adopted 311 mW, 32 kHz. It was suggested that a high pulse energy was used to obtain the through hole, and then a low pulse energy was used to modify the hole quality [65].

Considering that there is no detailed work to prove the basic relationship between picosecond laser and C_f_/SiC in the drilling process, Liu et al. investigated the influence of the two drilling modes (single ring line and helical lines scanning) and the influence of the energy density on the drilling performance from the aspects of drilling rate, mechanism, morphology feature, etc. A 532 nm, 6.8 ps, 100 kHz, 10 µm, 1–30 W picosecond laser was used drill C_f_/SiC with a thickness of 3 mm. For single ring line mode, the laser beam turned along the designed single ring with a speed of 40 rev/s, a number of 60, and a drilling time of 1.25 s. For helical lines scanning mode, the laser processing started from the center of the helical line ring and rotated along the arca of the helical line ring with a speed of 40 rev/s and a drilling time of 1.25 s. As shown in Figure 18c,d, the ripple substructure with a period of ~250 nm was uniformly formed under the two drilling modes. In addition, when the energy density was sufficiently high, such as 0.65 J/mm^2^ in the single ring line drilling and ~1.00 J/mm^2^ in the helical lines scanning, the rate of the increase in hole depth and width significantly dropped. The author believes that there are three causes: (a) the ejection of the ablated materials was limited to the high aspect ratio of the hole; (b) the debris consumed the laser energy; and (c) aspheric lens which always focused on the samples surface during drilling process, dispersed the energy beyond the beam focal plane, and affected the further processing [67]. Further study showed the effects of energy density and feed speed on the drilling performance in C_f_/SiC with different thicknesses of 2 and 3 mm, and the same drilling method could also be used to drill the SiC/SiC [20,68]. Additionally, in 2015, Zhang et al. used a 1 ps laser (1030 nm, 100 kHz, 20 µm, 8 W) to drill the C_f_/SiC ceramic to find the optimal parameters. The effects of different processing parameters, such as the scanning speed, helical line width and pitch, and the drilling time, on the surface morphology of drilled holes were analyzed. It was suggested that a smaller helical line spacing of 5 µm and a smaller helical line width of 8 µm were beneficial in terms of better drilling quality [69].

**Figure 19:**
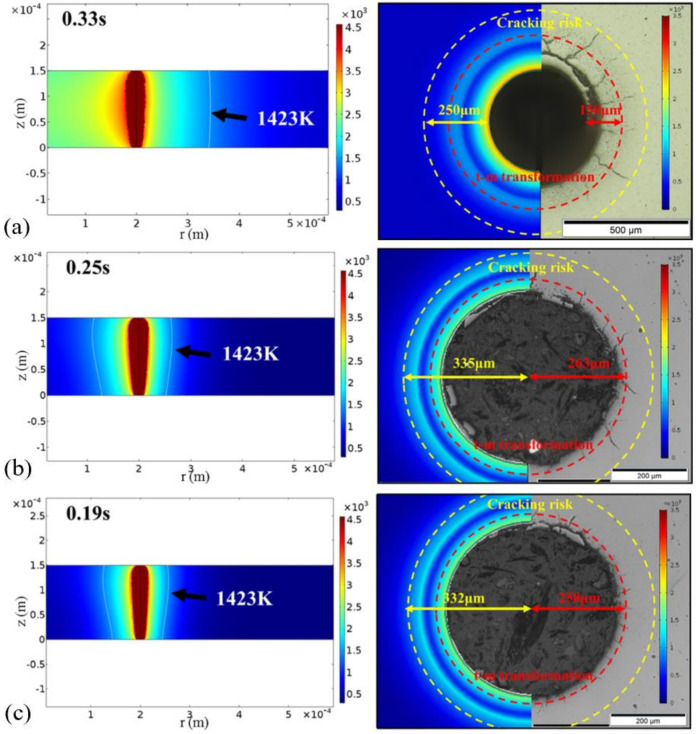
(**a**) Temperature distribution and the phase transformation at 0.33 s (50 W and 200 mm/s). Experiments and numerical simulations: (**b**) 42.5 W, 150 mm/s; and (**c**) 47.5 W, 200 mm/s. Reprinted with permission from ref. [70]. Copyright 2019 American Society of Mechanical Engineers.

Another important quality factor in ceramic drilling is cracks; few studies have comprehensively explained the occurrence of cracks through theoretical analysis. In 2019, Shen and Feng investigated crack behavior during the picosecond pulsed laser drilling of YSZ ceramic using acal model. A 1030 nm, 8 ps, 125 µJ, 400 kHz, 20 µm picosecond laser was used for the trepan drilling of YSZ with a thickness of 0.5 mm. As shown in Figure 19a,b, there was a phase transformation region with a diameter of approximately 700 µm, which led to there being fewer and finer cracks around the hole. In addition, it was proven that lower power and higher scanning velocity could improve the drilling efficiency and reduce the heat accumulation, as shown in Figure 19c. Higher laser power could improve the efficiency, but it also introduced more heat accumulation; higher scanning velocity improved the efficiency and reduced the heat accumulation [70].

**Figure 20:**
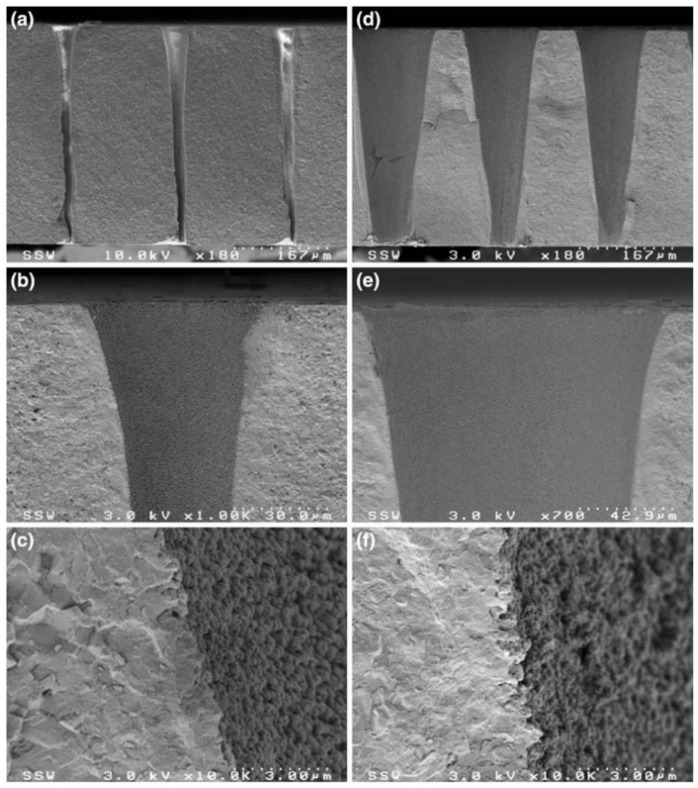
Cross-sections of the hole drilled by (**a**–**c**) percussion drilling; and (**d**–**f**) trepan drilling. Reprinted with permission from ref. [71]. Copyright 2009 Springer.

On the other hand, regarding research on femtosecond laser drilling in ceramic materials, in 2009, Li et al. compared the quality of the hole drilled by percussion and trepan methods. A 775 nm, 150 fs, 1 kHz, 1 W laser with a fluence ranging from 4 to 40 J/cm^2^ was used to drill the 96% Al_2_O_3_ substrates with a thickness of 0.39 and 0.48 mm. The results showed that the holes drilled by trepan method exhibited better dimensional accuracy and edge definition. The example of a 130 µm drilled hole (a 32 J/cm^2^, 10 mm/min) is shown in Figure 20. A thin recast layer of the order of ~30 nm was observed at the walls, possibly due to the melting and successive re-solidification of Al_2_O_3_ during laser processing. In addition, there were no cracks observed in this area during laser processing [71].

**Figure 21:**
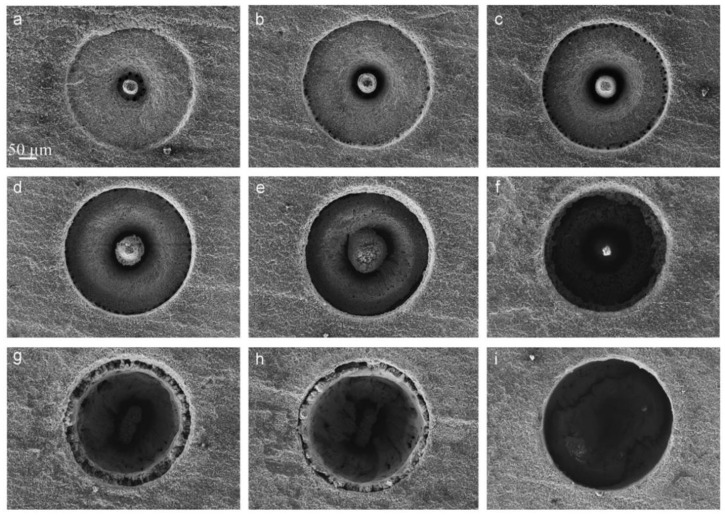
The blind holes drilled by the femtosecond laser with a repetition rate of (**a**–**i**) 2, 6, 10, 20, 40, 60, 80, 100, and 140 kHz. Reprinted with permission from ref. [73]. Copyright 2015 Elsevier.

To determine the optimal parameters for the femtosecond laser trepan drilling of ceramics, in 2010, Wang et al. studied the effects of the laser parameters on hole quality during the femtosecond laser drilling of Al_2_O_3_. A 775 nm, 150 fs, 800 mW, 1 kHz, 30 µm femtosecond laser was used for the trepan drilling of 99.6% Al_2_O_3_ with a thickness of 250/381/625 µm. The high-quality laser drilling of Al_2_O_3_ substrates with a clean surface, no cracks, no recast layer, and no delamination was demonstrated [72]. Additionally, in 2015, Zhang et al. studied the effects of a spiral number, processing time, laser fluence, and repetition rate on the hole quality. A 1030 nm, 230 fs, 1–500 kHz, 30 µm femtosecond laser was used for the helical drilling of the TiC with a thickness of 2 mm. For drilling a blind hole, it was found that the depth significantly increased when the laser fluence increased from 6.37 × 10^−1^ to 1.27 J/mm^2^, and with the further increase in laser fluence, the depth decreased. Additionally, it was found that the laser repetition rate had a vital effect on the energy deposition on the TiC surface, as shown in Figure 21. It was noteworthy that when the repetition rate was 140 kHz, the outer edge of the hole was clean, and the deposited layer became obviously thinner, which was possibly a result of the splash of molten materials out of the blind hole [73]. Additionally, the effects of the repetition rate on the circular rings’ drilling performance was further studied [73].

**Figure 22:**
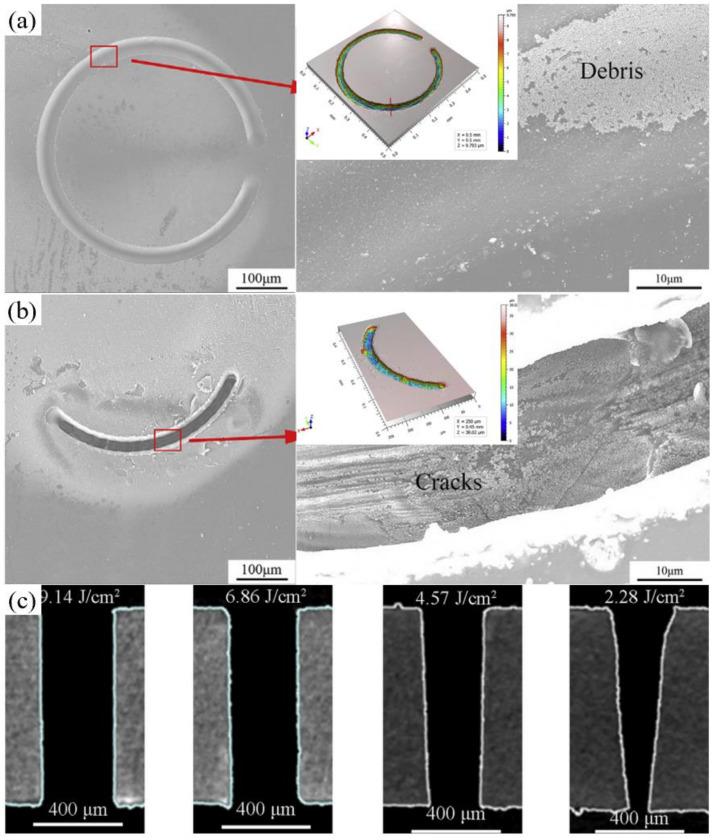
Single circular line drilling (2.28 J/cm^2^) at (**a**) 2400 rpm; and (**b**) 800 rpm; (**c**) cross-sections of the holes drilling at 9.14, 6.86, 4.57, and 2.28 J/cm^2^. Reprinted with permission from ref. [18]. Copyright 2020 Elsevier.

Recently, SiAlCN ceramics have been selected as substrates due to their low porosity and high oxidation/corrosion resistance at high temperatures, but their brittleness is the main disadvantage of its drilling. Chen et al. adopted the femtosecond laser to perform the precision processing of SiAlCN. By means of three different drilling methods—namely single circular lines, blind holes, and through holes—the influence of the laser fluence and rotational speed on the drilling quality of the SiAlCN was studied. A 1030 nm, 290 fs, 100 kHz was used to drill the SiAlCN with a thickness of 0.5–1.0 mm. A multilayer laser processing strategy with helical drilling was used to obtain the through holes, and the moving speed of the *Z* axis direction was 50 μm/s. Comparing the drilling results of the single circular lines, as shown in Figure 22a,b, it was apparent that a rotational speed of 2400 rpm was preferred for performing drilling considering the cracks, HAZ, debris, width, and depth of the ablated circular lines. Finally, as shown in Figure 22c, it is recommended to use a rotation speed of 2400 rpm and laser fluences of 5.72–8.00 J/cm^2^ to ensure the quality of the drilling [18].

## 6. Liquid-Assisted Laser Drilling of Ceramic

### 6.1. Drilling Mechanism

Prior to 1992, Mueller et al. explored the physical mechanism of the interaction between laser and water. A CW CO_2_ laser was used to irradiate the water, and it was proven that the laser energy was efficiently absorbed by the water and rapidly vaporized the water. Additionally, it was proposed that the vaporization of the water would form a cone-shaped hole shape, and lead to many bubbles in the water [74]. Since the heat conductivity of water is better than that of air, and the laser heat will be dissipated into the water, the water-assisted laser drilling will cool the sample and help obtain good drilling quality. In this context, in 2004, Lu et al. studied the mechanism of laser drilling of metal target with water-assisted laser drilling. It was suggested in the water environment, the laser-produced plasma with a higher pressure would create a smaller sized plasma, and thus the water-assisted laser drilling required higher laser fluence for drilling the target [75].

**Figure 23:**
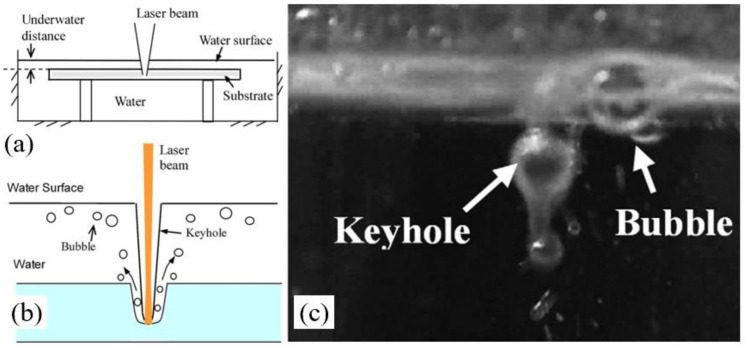
(**a**) Schematic diagram of underwater laser drilling; (**b**) interaction between the laser, water, and substrate; and (**c**) test results of the keyhole and the bubbles. Reprinted with permission from ref. [76]. Copyright 2009 Elsevier.

In 2009, Tsau and Li applied the laser-assisted drilling method for drilling the ceramic. A 10.6 μm pulsed CO_2_ laser with a diameter of 130 μm was used to drill the 96% Al_2_O_3_ ceramic with a thickness of 0.63 mm. Figure 23a is the schematic diagram of underwater laser drilling. It was pointed out that the thickness of the water had a great influence on the drilling performance considering the dynamic physical properties of the laser and water. Excessive water film thickness would cause a large amount of laser energy loss, while an excessively thin water film brings an insufficient cooling effect. Finally, an optimal water film parameter was determined to be that of 1 mm under the water. Figure 23b shows the schematic diagram of the interaction between the laser and the substrate placed in the water environment. When the laser was irradiated on the water surface, part of the laser energy would be absorbed by the water, and the other part would penetrate the water and finally be absorbed by the substrate. Figure 23c shows a figure detected when the laser interacted with the water. A keyhole and bubbles could be found and it was proposed that the bubbles would influence the keyhole shape. The authors proved that the water-assisted laser drilling could be used to percussion drill the array-holes of the order of 100 μm in diameter, and to trepan drill large holes with a diameter greater than 10 mm [76].

Since laser processing in water is enhanced by the better laser–matter interaction, which limits heat accumulation, and removes debris to avoid re-deposition, it has been proven that it the mechanical impact related to the formation and the collapse of bubbles of evaporated material [77,78,79,80] can be widely used in the laser drilling of ceramic materials, including long and femtosecond pulsed laser.

### 6.2. Long or Short Pulsed Laser Drilling

In 2014, Lu and Yuan studied the effects of the auxiliary gas pressure, pulse energy, and repetition rate on the hole quality when using a low-pressure water jet. A 1064 nm, 0.1–10 ms, 500 W, 0–100 Hz millisecond laser was used to drill the Al_2_O_3_ with a thickness of ~1 mm. The auxiliary gas was coaxial with the laser beam with a pressure from 0 to 0.5 MPa. The speed of the water jet could be adjusted from 0 to 28 m/s with a nozzle diameter of 0.7 mm. It was concluded that the significant role of the auxiliary gas was blowing water film, and the slag removal was the result of the water jet erosion. The desired quality could be obtained at an auxiliary gas pressure of 0.3–0.4 MPa and a repetition rate of 45 Hz [81]. In 2018, Li et al. proved that the static water-assisted millisecond laser drilling method could significantly improve the hole quality when compared with the drilling in air. It was shown that the taper angle, slag, recast layer, and cracks of the holes were remarkably reduced [82]. Further work also studied the influence of water and bubbles on the drilling quality in the water environment. It was suggested that the collapse and rupture of the bubbles produced shock and vibration, causing the recast layer on the inner wall of the hole to fall off [83]. In addition, it has been proven that the presence of a liquid medium helped control the HAZ and the taper angle on the smart ceramic substrate (piezoelectric ceramic) which was also drilled by the CW CO_2_ [84].

**Figure 24:**
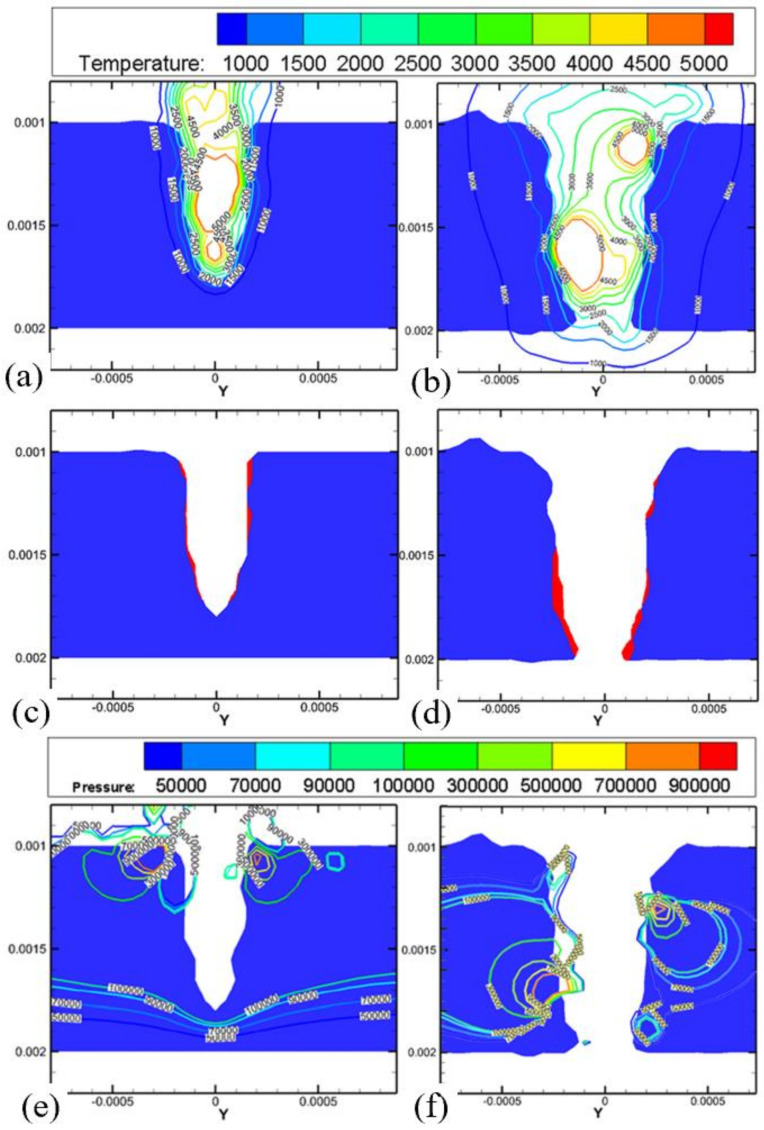
Temperature simulation of the drilling (**a**) underwater and (**b**) in air; simulation of the molten zone (**c**) underwater and (**d**) in air; and pressure simulation of the drilling (**e**) underwater and (**f**) in air. Reprinted with permission from ref. [85]. Copyright 2017 AMSE.

Experiments have proven that the water-assisted drilling method could significantly improve the drilling quality, however, there are few theoretical models which can analyze this in detail; therefore, in 2017, Shen et al. developed a 2D transient model to simulate the underwater laser drilling of Al_2_O_3_. The distributions of the temperature, pressure, and velocity during the drilling process were examined. The numerical results show that the underwater-drilled hole with a smaller taper was obtained compared with that in air, which was attributed to the recoil pressure, higher specific heat capacity, and heat transfer coefficient of water. Figure 24 shows the distributions of the temperature and pressure at the irradiation of the third laser pulse. The depth of the hole drilled under water was approximately 700 μm (Figure 24a) and the hole was nearly finished in the air environment (Figure 24b). Additionally, the lesser amount of melt demonstrated that the water-assisted drilling could reduce the HAZ and recast layer, as shown in Figure 24c,d. The distribution of pressure shows that regardless of being underwater or in air, the position of the high pressure and the value of pressure were similar. The present study confirms that water-assisted laser drilling is an effective way to reduce the ejection on the top surface and the taper angle of the hole, while drilling efficiency is reduced [85].

On the other hand, regarding the research into liquid-assisted nanosecond laser drilling, Wee et al. investigated the laser drilling of the SiC wafer in air, underwater, and in methanol. A 355 nm, 24–35 ns, 30–85 kHz, 46–150 μJ, 25 μm nanosecond laser was used to drill the SiC with a thickness of 0.5–1.5 mm. It was shown that the laser drilling of SiC under methanol was able to drill holes with a relatively cleaner and smoother surface. It was suggested that the relatively fast evaporation of methanol could carry away the ablated particles of the SiC at a relatively higher rate. As a result, the material redeposition was minimized and oxidation effects were also reduced [86]. Additionally, in 2014, Iwatani analyzed the effects of water film thickness, laser fluence, pulse number, and focus position on the water-assisted laser drilling of SiC. A 1064 nm, 6 ns, 4.72 W nanosecond laser was used to drill the SiC with a thickness of 0.34 mm. It was shown that water-assisted laser drilling could create holes without debris, HAZ and cracks, and the optimal parameters were suggested to be a laser fluence of less than 10 J/cm^2^ and a water thickness of 1 mm [87].

Moreover, Garcia-Giron et al. used a new liquid (ethylene glycol) to assist the laser drilling of Al_2_O_3_, 8YSZ and glass–ceramic samples, and compared the drilling with the assistance of water. The density and viscosity of ethylene glycol are 11 times and 18 times that of water, respectively. This hardly generates shock waves and cavitation bubbles, resulting in a very low and attenuated mechanical ablation process. Therefore, the results show that only the most brittle materials (glass–ceramic) could be processed under the optimal parameters. In addition, it was also proven that under the assistance of the water, the ablation rates of glass–ceramics, 8YSZ, and Al_2_O_3_ were 3.88, 1.76, and 26.06 times higher than that drilled in air, respectively [88].

**Figure 25:**
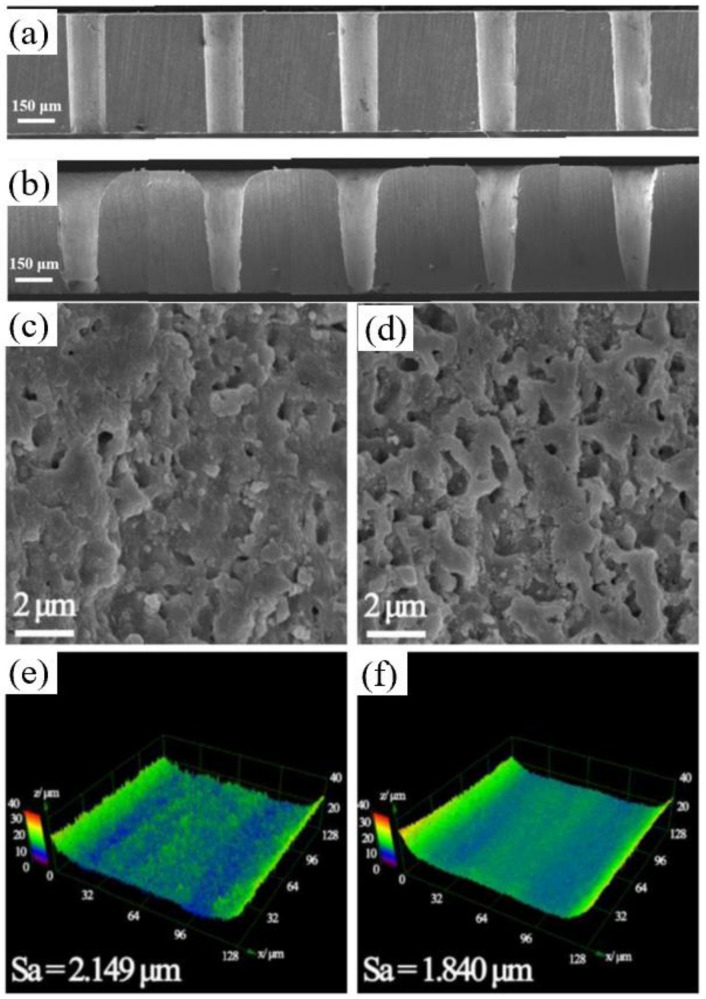
Cross-section of the holes drilled in (**a**) air and (**b**) water with a different scanning speed. Micrographs and roughness of the hole cross-section in (**c**,**e**) air and (**d**,**f**) water. Reprinted with permission from ref. [89]. Copyright 2018 Elsevier.

In addition to percussion drilling with the assistance of water, the trepan drilling has also been studied. In 2018, Chen et al. used a 532 nm, 6 ns nanosecond laser to drill the Si_3_N_4_ with a thickness of 0.5 mm at 2 mm underwater (0.4 µm pitch, 100–800 mm/s scanning speed, 150 µm diameter and 20 times for drilling a hole). In contrast to the percussion drilling described above, the water-assisted laser made the taper of the hole larger than that in the air, as shown in Figure 25a,b. However, the taper angles decreased with the increasing scanning speed underwater. In addition, the smoother surface on the cross-section underwater, as shown in Figure 25c,d, could be attributed to the slighter chemical reactions and effects of higher pressures and shock waves, which effectively removed the adhered debris and molten materials [89].

### 6.3. Ultrafast Pulsed Laser Drilling

To achieve deeper holes during the ultrafast laser drilling of ceramic, removing the ablation debris becomes a critical problem. A common practice to control ablation debris is to use an inert gas flow over the laser ablation region, which is intended to prevent oxidation reactions, cooling the substrate, and flushing the ablated material away. Unfortunately, as the geometric size of the laser focus decreases and the instantaneous laser power increases, this technique becomes less effective. At this point, the water-assisted laser helps further carry away the ablation debris and reduce the ablated material redeposition.

**Figure 26:**
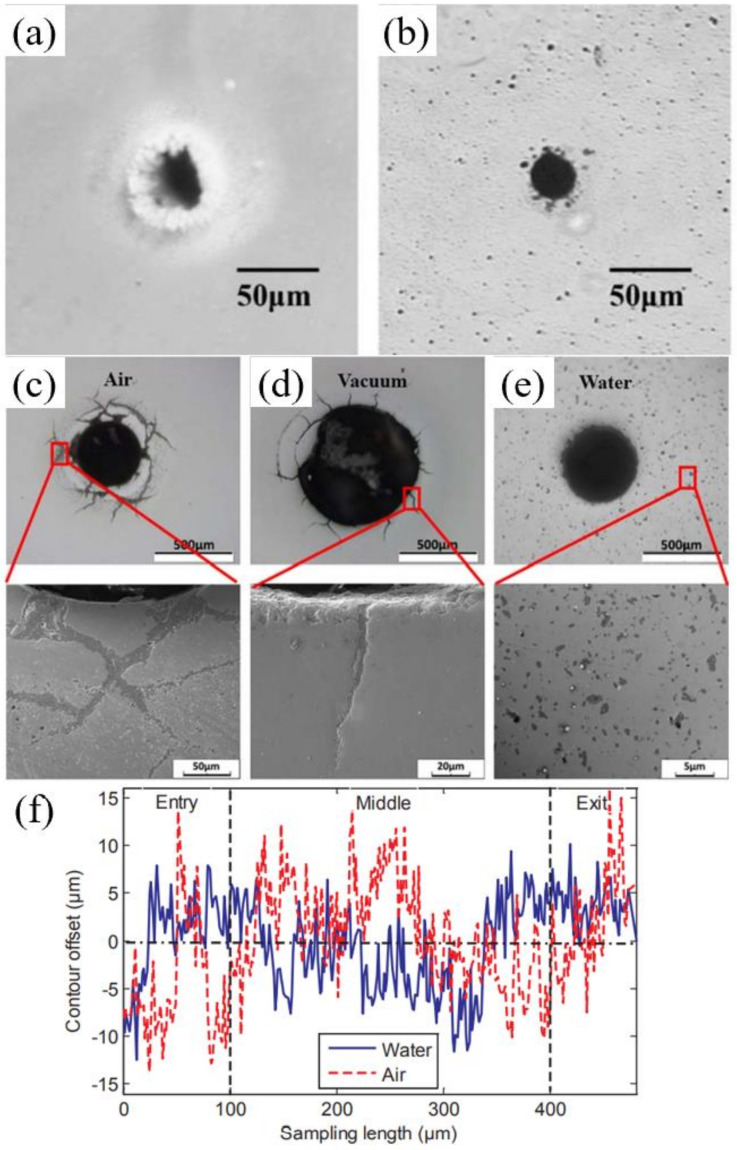
Upper surface of the hole drilled in (**a**) air and (**b**) alcohol. Reprinted with permission from ref. [90]. Copyright 2009 Elsevier. Cracks of the hole drilled in (**c**) air, (**d**) vacuum, and (**e**) water Reprinted with permission from ref. [92]. Copyright 2004 Elsevier. (**f**) Typical profile along the hole. Reprinted with permission from ref. [16]. Copyright 2019 Elsevier.

In 2009, Li et al. used alcohol to improve SiC drilling under the irradiation of a femtosecond laser. An 800 nm, 30 fs, 1 kHz femtosecond laser was used to drill the SiC with a thickness of 250 µm (50 µm/s scan speed, 40 µm diameter, 3.1 GW/cm^2^ peak power density, and 3 s for drilling a hole). For laser drilling in air, it is difficult to increase the ablation depth because of the accumulation of debris and redeposition of ablated material. On the contrary, deeper through holes could be easily fabricated with alcohol assistance. Additionally, it is worth noting that there was no indication of typical thermal damage around the hole. A nearly circular shape with a diameter approximately 40 µm was produced at the top side, as shown in Figure 26a,b, as the up side of the wafer displayed very little surrounding debris [90]. On the other hand, it has been proven that an acid etching method could also be used to improve the performance of femtosecond laser drilling [14,91].

Then, in 2019, Shen et al. studied the environmental effect (three different environments: air, water, and vacuum) on the cracking behavior of YSZ during picosecond laser drilling. A 1030 nm, 8 ps, 125 µJ, 400 kHz, 20 µm picosecond laser was used to drill the YSZ with a thickness of 0.5 mm (400 µm diameter). The crack patterns of YSZ after laser drillings in different environments are shown in Figure 26c–e. It can be seen that the hole diameter obtained in vacuum was much bigger than the other two, and no cracks occurred during water-assisted drilling. It was proposed that reducing the thermal stress and t-m transformation might be an efficient way to eliminate cracks during laser drilling [92]. Further work analyzed the effects of process parameters on hole quality to obtain the optimized parameters by using a multi-objective optimization method. It was shown that the most important factors affecting the overall quality were the scanning speed and the scanning number, and it was proven that the water environment was conducive to reducing the cracks and surface roughness—as shown in Figure 26f [16]. In addition, the optimal parameters to drill the Al_2_O_3_ ceramic and SiC ceramic were also systematically studied by Ren et al. [13,93]. Moreover, the crystal cleavage, periodic nanostructure and surface modification of SiC ablated by the femtosecond laser in different media (air, water, HF) was also studied by Wu et al. [22].

**Figure 27:**
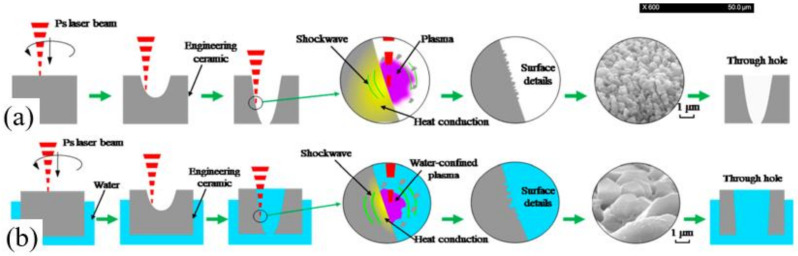
(**a**) schematic of the through hole formation mechanism in the direct laser trepan of Al_2_O_3_; and (**b**) schematic of the through hole formation mechanism in the SWILT of Al_2_O_3_ [94].

In the above related studies in liquid-assisted drilling, the samples were completely placed underwater, which sometimes gives the upper surface of the hole an obvious taper, as shown in Figure 25b. In 2019, Ma et al. adopted a completely different approach, which was called a semi-water-immersed laser trepan drilling method (SWILT). The Al_2_O_3_ substrate was partially immersed in water and the bottom of the substrate was immersed in water. A 1064 nm, 12 ps, 0.2–1 MHz, 20 µm, 70 W picosecond laser was used to drill the 95% Al_2_O_3_ with a thickness of 0.6 mm. Using the optimized parameters, a straight through hole with an average diameter of approximately 79.2 µm and an aspect ratio ~8 could be repeatably produced. In addition, compared with the results of direct trepan drilling, SWILT generated through holes with the smallest taper angle and straighter sidewalls, while the ablation surface quality was relatively rough. The formation mechanisms of the two different drilling methods are shown in Figure 27a,b. In the case of direct trepan drilling without the assistance of water, when the incident laser beam interacted with the target, a blind hole was formed, and the target was removed in a purely thermal-dynamic manner, resulting in a deepening of the transition hole until the through hole was finally formed. The re-solidification of the residual melt after laser pulse heating might be densely distributed sub-micron bumps on the sidewall surface. In contrast, for SWILT, once the Al_2_O_3_ substrate was drilled through, due to the siphon effect, water was pumped up and filled the holes, resulting in a laser-induced break down in the water. This was followed by water-confined plasma and its associated strengthened mechanical effects, which might cause the material removal of molten or softened layers, leading to an increased material-removal rate, especially in tighter spaces [94].

## 7. Combined Pulse Laser Drilling of Ceramic

**Figure 28:**
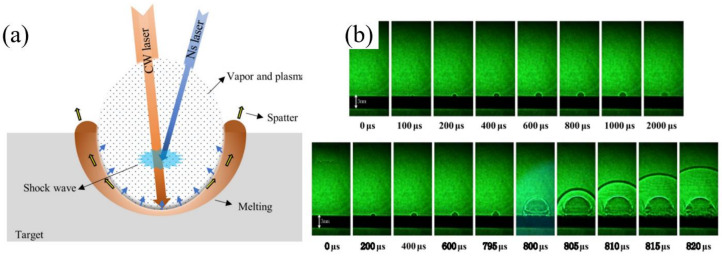
(**a**) Laser–matter interaction of the CPL. Reprinted with permission from ref. [96]. Copyright 2018 Optica. (**b**) Plasma plume expansion evolutions for the single millisecond laser (**upper**) and CPL (**lower**) Reprinted with permission from ref. [97]. Copyright 1975 AIP.

The possibility of processing material using the CPL method that combines the CW laser and nanosecond pulsed laser, demonstrated by Fox in 1975, has attracted attention in a wide range of areas related to academic research and engineering [95]. The basic idea of using the CPL to process materials is to improve the MRR by controlling the temporal or spatial profiles of the CPL to meet the different laser energy requirements during the laser–matter interaction. Thus, the CPL method drives the exploration to optimize the laser–matter interaction, and began the race towards these and many other attractive goals. Figure 28a shows a schematic diagram of the laser–matter interaction of the CPL. The introduction of the nanosecond laser pulse can help form a high recoil pressure or a high shock wave pressure, which can be used to facilitate the melt expulsion during the long pulsed laser irradiation [96]. The comparison of a typical plasma plume expansion evolution of the CPL drilling with the single millisecond laser is shown in Figure 28b, in which a shock wave generated on the target surface creates a splashing phenomenon and the expansion speed of the plasma plume reaches 600 m/s—which led to a 9-fold increase in the ablation depth of the aluminum alloy [97]. To date, the ideas of developing CPL have been explored for improving the efficiency and quality of laser processing in a wealth of applications including laser ablation [98,99,100,101,102], structuring [103,104,105,106], drilling [107,108,109,110], and welding [111,112,113,114].

**Figure 29:**
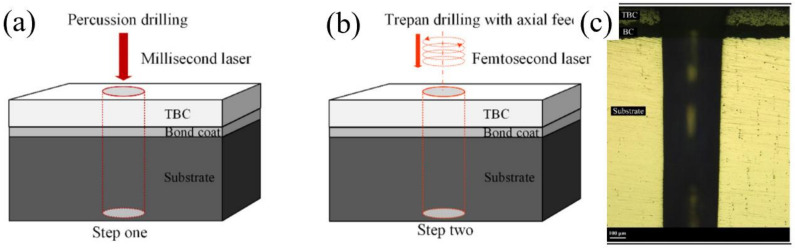
(**a**) Step one and (**b**) step two of the ms-fs CPL drilling; (**c**) the hole drilled by the two-step CPL. Reprinted with permission from ref. [115]. Copyright 2019 Elsevier.

One typical method to make full use of the advantages of the ultrafast laser in the CPL processing is to use the ultrafast laser processing as the finishing method to improve the drilling quality. An interesting experiment was reported by Wang et al. in 2019 which we found worked extremely well when used in the high-efficiency and high-quality drilling of structural ceramics: a two-step CPL approach to drill the thermal barrier coated nickel base alloys. As shown in Figure 29a,b, a through hole was initially created by a modulated millisecond laser and was then refined by a trepan femtosecond laser. The results showed that drilling through the hole by the millisecond laser took approximately 0.4 s and refining the recast layer and delamination cracks by the femtosecond laser took 5 min, and finally, the recast layer and delamination cracks were completely eliminated, as shown in Figure 29c. It can be seen that the two-step CPL processing method was proven to be effective in decreasing the thermal effect of long pulse laser drilling, so as to fully improve the processing quality. However, due to the speed limit of the femtosecond laser trepan mode, the processing efficiency of the femtosecond laser is still limited, which needs to be further solved [115].

As mentioned above, CPL processing technology has been extensively studied in the processing of metal materials; however, few studies have been reported for drilling ceramics. In fact, ceramic materials are typically hard and brittle materials, which are easy to cause cracks, taper and other defects due to the high heat input of the laser energy during the drilling. Since 2001, although the CPL technology is not directly used in ceramic drilling, researchers have used the CPL processing technology to decrease the thermal stress and modify the processing surface quality of the ceramic [116,117,118,119]. In addition, in order to obtain a crack-free, dense laser-treated zone, flame-assisted [120], plasma heating technique [121], and temperature electric furnace [122] have also been used obtain a crack-free, dense laser-treated zone.

**Figure 30:**
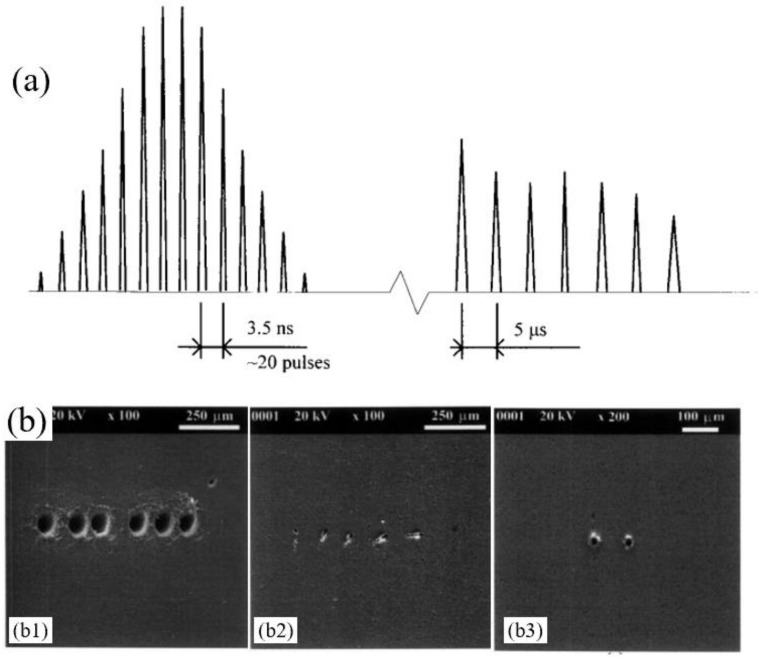
(**a**) The picosecond–nanosecond CPL; (**b1**) upper and (**b2**) lower surface holes drilled by the laser with different polarization directions; (**b3**) holes drilled by circular polarized beam. Reprinted with permission from ref. [118]. Copyright 1999 Springer.

In fact, in the papers related to the CPL drilling of ceramic materials, as early as 1999, Klimentov et al. proposed a two-step picosecond–nanosecond CPL drilling method for AlN ceramic. Figure 30a shows the schematic temporal profile of the picosecond–nanosecond CPL. The CPL consisted of an initial train of picosecond pulses with an axial period of 3.5 ns which, after a time delay of 10 μs, was followed by a sequence of 10–20 Q-switched 200 ns pulses during a period of 5–7 μs. The high-fluence picosecond pulses were used to ablate a modify area that helped improve absorption of the followed nanosecond laser pulses. As shown in Figure 30b, the pronounced polarization dependence observed in the special tests implied linear and circular polarized beams. This dependence manifested itself in the output hole shape. While the entrance hole was round, disregarding the incident beam polarization [118]. In addition, in 2009, D. P. Hand et al. proposed a two-step CPL drilling method for drilling the ZrO_2_ ceramic. A high-energy millisecond laser was used for drilling an initial hole with a rough surface quality while it had a high processing speed, and then a picosecond laser was used to provide the “finishing cut’’, generating fine-scale features with minimal thermal impact on the ceramic. It was proved that the ms-ps CPL drilling method was an efficient process for the generation of tooth restorations [123].

**Figure 31:**
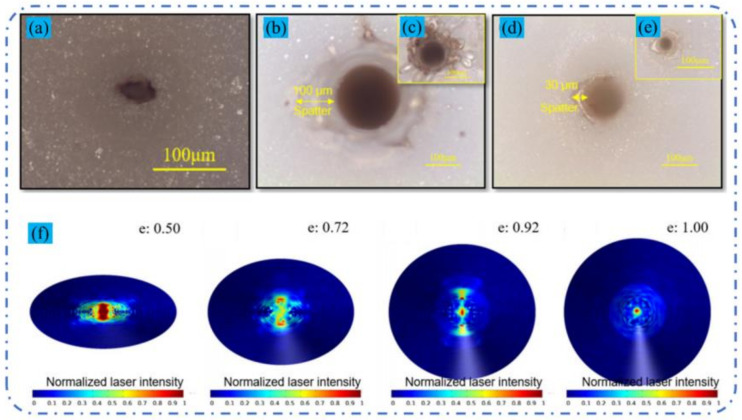
Hole drilled by (**a**) the single nanosecond laser; (**b**,**c**) the single millisecond laser; and (**d**,**e**) the ns/ms CPL. (**f**)The top view shows the simulation laser density distributed on the inner wall of the keyhole with a minor axes of 12.5, 18.0, 23.0, 25.0 μm [124].

Recently, Jia et al. tried two different CPL methods for drilling the Al_2_O_3_ substrate, named ns/ms CPL (one ms pulse and one ns pulse train with 6 short pulses were output synchronously) and ns-ms CPL (an initial train of nanosecond pulses which, after a time delay of ~1 s, was followed by a sequence of millisecond pulses). The millisecond laser was a 1070 nm, 800 W, 2 ms, 50 Hz, and 50 μm gated CW laser, and the parameters of the nanosecond laser were 1064 nm, 4 mJ, 17 ns, 3 kHz, an ellipse focused beam. The results showed that the single nanosecond laser could only ablate a blind hole with a depth of 20 μm under the irradiation of 60 nanosecond laser pulses, and the single millisecond laser with a peak power of 700 W could drill through the target under the irradiation of 10 pulses. However, the ns/ms CPL with a 60 W peak power millisecond laser could drill through the target and obtain a well quality, as shown in Figure 31a–e. The authors believed that the keyhole ablated by the nanosecond laser improved the absorptivity of the original sample surface through the multi-reflection of the hole wall, and the high-pressure plasma shock wave induced by the nanosecond laser also increased the melt ejection; thus, good drilling performance was achieved. Moreover, the keyhole ablated by the nanosecond laser was elliptical due to the off-axis distortion of the system, however, the circularity of the hole drilled by the CPL was not affected because the laser was actively homogenized in the hole through multi-reflection, as shown in Figure 31f [124]. Then, a series of experiments was used to further confirm this view [23,125,126].

## 8. Conclusions and Outlook

The laser drilling method was demonstrated to be a practical tool for high-quality drilling in ceramics, and it is widely used in the processing of a variety of structural ceramics such as Al_2_O_3_, SiC, Si_3_N_4_, AlN, YSZ, ZTA, 2.5D C_f_/SiC composite, C_f_/SiC composites, SiC/SiC composite. This review introduces the recent progress made in the laser drilling of structural ceramics. Future research is expected to pay more attention to improving the performance of the laser drilling of structural ceramics, establishing a more realistic mathematical model to simulate the physics with more accuracy via the assistance of software and developing multi-assisted techniques. Here, we will briefly summarize the opportunities and challenges of ceramic drilling-related research.

First, to improve the drilling performance of different lasers, the sub-microsecond laser will become the focus of future research since it has great potential in industrial applications where a relatively low precision requirement is acceptable. It has been proven that high-quality holes with a diameter of 100 μm at a drilling speed of 66 holes/s was achieved by the use of a sub-microsecond pulsed thin disk laser [53]. On the other hand, for applications with high processing quality requirements, the research and application of an ultrafast laser has become increasingly developed. Since the heat accumulation in the ultrafast laser drilling area is very small, it is expected to eliminate the recast layer and microcracks in the hole wall and realize the processing of high-quality large-area holes.

In addition, the theoretical research into the application of auxiliary methods such as liquid-assisted laser drilling method and the CPL drilling method will become another new research focus. Taking the combination of CW and femtosecond laser as an example, under the drilling of the CW/femtosecond CPL method, the pulse durations of the CW laser and femtosecond laser belong to different time scales, and the simulation across time scales will be a brand new challenge. Moreover, the pump-probe shadowgraph method is now greatly helpful to understand the transient changes in the materials during the drilling, and can detect changes in the order of femtoseconds. Therefore, further exploration of the plasma evolution and the sputtering removal process during the CPL drilling will help us deepen the understanding of the internal physical mechanism of the laser–matter interaction. Therefore, the performance of micro-hole drilling can be further regulated to obtain highly efficient and high-quality drilling.

Moreover, hybrid laser drilling methods can also effectively improve the quality of ceramic drilling and accurately regulate hole morphology, such as in chemical etching hybrid laser processing, and ultrasonic vibration laser hybrid laser [127].

## Figures and Tables

**Table 1:** The removal mechanisms of the different ceramics [30].

Ceramic	Melting	Evaporation	Dissociation	Dissociation Process
Al_2_O_3_	√	√	√	Al_2_O_3_→2Al_(l)_ + 3/2O_2(g)_
Si_3_N_4_	√	√	√	Si_3_N_4_→3Si_(l)_ + 2N_2(g)_
SiC	√	√	√	SiC→Si_(l)_ + C_(s)_
MgO	√	√	√	MgO→Mg_(s)_ + O_(g)_

## Data Availability

Not applicable.
